# CHI3L1: a key driver in gastritis-to-cancer transformation

**DOI:** 10.1186/s12967-025-06352-2

**Published:** 2025-03-19

**Authors:** Tao Li, Huizhong Jiang, Yucheng Gong, Mengting Liao, Yuanping Jia, Jiena Chen, Ming Dai, Yinan Yan, Xinyu Lu, Runhua Chen, Yuan Li, Yan Chen, Jie Lin, Yicong Li, Xia Ding

**Affiliations:** 1 School of Traditional Chinese Medicine, Beijing University of Chinese Medicine, Beijing, 100029 China; 2 Dongzhimen Hospital, Beijing University of Chinese Medicine, Beijing, 100700 China; 3 MOE Key Laboratory of Membraneless Organelle and Cellular Dynamics, Hefei National Laboratory for Physical Sciences at the Microscale, University of Science and Technology of China, Hefei, 230027 China; 4 Dongfang Hospital, Beijing University of Chinese Medicine, Beijing, 100078 China; 5 National Institute of Traditional Chinese Medicine Constitution and Preventive Treatment of Diseases, Beijing University of Chinese Medicine, Beijing, 100029 China; 6 Research Center for Spleen and Stomach Diseases of Traditional Chinese Medicine, Beijing University of Chinese Medicine, Beijing, 100029 China

**Keywords:** CHI3L1, Gastritis-to-cancer transformation, Biomarkers, Diagnostics, Bioinformatics, Gastric precancerous lesions, CD44-β-catenin pathway

## Abstract

**Background:**

Gastric cancer, recognized as one of the most lethal malignancies globally, progresses through a complex, multi-stage development. Elucidating the pathogenic mechanisms behind gastric carcinogenesis and identifying early diagnostic biomarkers are pivotal for decreasing the prevalence of gastric cancer.

**Methods:**

Using datasets on gastric cancer and its transformation from gastritis, we employed machine learning to create an early diagnostic model, identifying key genes and evaluating accuracy. We prioritized genes in the gastritis-to-cancer progression, identifying a central driver gene. Pathway analysis revealed its transformation role. Tissue microarrays and rat models validated the driver genes and networks, confirmed in cell and organoid models. We also identified cell types secreting CHI3L1 using single-cell RNA sequencing and multiplex immunohistochemistry, exploring their prognostic significance.

**Results:**

We identified 12 driver genes potentially involved in the gastritis-to-cancer transformation, with CHI3L1, MMP12, CXCL6, IDO1, and CCL20 emerging as the top five genes via a early gastric cancer diagnostic model. CHI3L1 was pinpointed as the central driver across the gastritis-to-cancer spectrum, with its upregulation, along with CD44, β-catenin, and c-Myc, noted in gastric precancerous lesions. In vitro and organoid studies revealed CHI3L1’s role in activating the CD44-β-catenin pathway to induce malignancy. Furthermore, our findings indicate that fibroblasts and dendritic cells are the principal sources of CHI3L1 secretion, a factor that is associated with poor prognosis in gastric cancer.

**Conclusions:**

This study highlights CHI3L1 as a key gene driving the progression from gastritis to gastric cancer, primarily by activating the CD44-β-catenin pathway, which enhances malignant cell traits. CHI3L1 is mainly secreted by fibroblasts and dendritic cells, and its high levels are linked to poor gastric cancer prognosis.

**Supplementary Information:**

The online version contains supplementary material available at 10.1186/s12967-025-06352-2.

## Introduction

Gastric cancer (GC) is recognized as one of the most lethal malignancies worldwide, with its pathogenesis following a sequential multistage process that includes chronic non-atrophic gastritis, chronic atrophic gastritis, intestinal metaplasia, dysplasia, and ultimately carcinoma [1]. Understanding the pathological mechanisms underlying gastric carcinogenesis and identifying early diagnostic biomarkers are crucial for reducing the incidence of GC [2]. Currently, the principal driver genes and the fundamental pathological mechanisms involved in the progression from gastritis to GC are not sufficiently understood [3]. Therefore, it is crucial to analyze GC datasets in conjunction with datasets pertaining to the transformation from gastritis to cancer in order to identify the key driver genes implicated in this transition [4, 5]. This analytical approach facilitates the development of a diagnostic model that assists in the early detection of GC, thereby enabling timely intervention.

The initiation and development of GC are intricately linked to inflammatory responses, characterized by transitions from gastritis to carcinogenesis. Chronic inflammation orchestrates a variety of cellular outcomes, including the loss of epithelial cell polarity, disruption of intercellular junctions, and detachment from the basement membrane [6–8]. These changes precipitate epithelial-mesenchymal transition (EMT), migration, and invasion [9]. Gastric precancerous lesions (GPL), such as intestinal metaplasia and dysplasia, constitute pivotal stages in the malignant transformation from chronic gastritis to GC [10]. Gastric epithelial cells, when stimulated by various cytokines, undergo transdifferentiation into intestinal and dysplastic phenotypes, a process that is accompanied by increased proliferation [11]. Elucidating the molecular mechanisms underlying abnormal cell proliferation and differentiation in the premalignant phase is crucial for understanding the pathogenesis of GC and identifying potential therapeutic targets.

A substantial body of research has employed advanced sequencing technologies, including genomics and transcriptome sequencing, to identify potential biomarkers linked to the diagnosis and prognosis of GC, such as COL4A1, Chitinase-3-like protein-1 (CHI3L1), and various miRNAs. Nonetheless, there is a relative scarcity of studies concentrating on the gastritis to cancer transformation [12–14]. In this study, we employed datasets to investigate biomarkers throughout the pathological progression from inflammation to cancer. Utilizing bioinformatics and machine learning techniques, we developed a diagnostic model for GC, identified key driver genes, and determined CHI3L1 as the central driver gene in the transformation. To further investigate the underlying mechanisms, we developed a rodent model of gastric mucosal carcinogenesis and assessed the expression levels of key driver genes, focusing on the CHI3L1 related pathway. Subsequently, we generated human GPL cells and organoids to validate the role of CHI3L1 in regulating downstream pathway and promoting malignancy. Furthermore, we conducted an analysis of the specific cell types responsible for the secretion of CHI3L1 and evaluated the correlation between its expression levels and the prognosis of GC.

## Materials and methods

### Gene expression profiling data acquisition

Obtain the GPL dataset (GSE55696) [15] and GC datasets (GSE66229 [16], GSE79973 [17]) from the Gene Expression Omnibus Database (GEO). The GSE55696 dataset contains 20 chronic gastritis (CG) samples, 19 low-grade intraepithelial neoplasia (LGIN) samples, 20 high-grade intraepithelial neoplasia (HGIN) samples, and 19 early gastric cancer (EGC) samples. The GSE66229 dataset contains 100 normal samples and 300 GC samples. The GSE79973 dataset serves as the validation set, consisting of 10 normal samples and 10 GC samples. Preprocess the three datasets separately and use the “limma” package [18] for standardization and normalization for quality control. Samples were merged by “ComBat” in the “sva” package with the algorithm for batch correction.

### Screening of diagnostic biomarkers for the transformation of gastritis to cancer

We aimed to identify genes associated with the transformation characteristics of chronic gastritis from inflammation to cancer. Based on the literature and previous research [19], we conducted Weighted Gene Co-Expression Network Analysis (WGCNA) analysis on the GSE55696 dataset containing gastritis to cancer transformation samples (CG-LGIN-HGIN-EGC) [20]. Use a soft threshold of 1–20 for topology calculation to determine the optimal soft threshold. Transform the relationship matrix into an adjacency matrix, and then further transform it into a topological overlap matrix (TOM). To classify TOM-based modules, use the average linkage hierarchical clustering method, with each module containing no less than 60 genes. Similar modules were subsequently merged. Finally, the Pearson method was used to calculate the correlation between the merged module and the features of inflammatory cancer transformation disease, and the module with the strongest positive correlation with the features was selected as the core module. In addition, we define gene significance (GS) as a measure of the association between a single gene and a target trait, and module membership (MM) as a measure of the correlation between gene expression profiles and the principal components of a given module. Simultaneously, we performed differential gene expression (DEGs) analysis on GSE66229 data using the “limma” package, comparing the differentially expressed genes between normal samples and GC tissue samples. The criteria were defined as P.adj.value < 0.05 and logFC = 0.585. LogFC > 0.585 is defined as an up gene, logFC < 0.585 is defined as a down gene, and the remaining genes are not included in subsequent studies. Intersection of WGCNA and DEGs to obtain candidate genes for diagnostic biomarkers of inflammatory-cancer transformation.

### Gene ontology (GO) and Kyoto encyclopedia of genes and genomes (KEGG) enrichment analysis and gene expression

The “clusterProfiler 4.12.6” package [21] was utilized for enrichment analysis of diagnostic biomarkers, employing both GO and the KEGG databases. We use GO to annotate the biological processes, molecular functions, and cellular components of genes. Use KEGG to annotate gene pathways. When P.adj.value < 0.05, enrichment has statistical significance. For candidate genes, use the limma package to extract expression levels for intergroup comparison differences.

### Construct the transformation progression stage model

In order to clarify the role of gastritis-to-cancer biomarkers in the diagnosis of GC, we constructed a GC diagnostic model based on random forest (RF) [22], support vector machine (SVM) [23], extreme gradient boosting (XGBoost) [24], and generalized linear model (GLM) [25]. The “DALEX” package [26] was used to explain the model, calculate the residual reverse accumulation of the four methods, and compare the Receiver operating characteristic (ROC) curves of the model’s diagnostic efficiency. The samples were randomly divided into the training and validation groups in a 7:3 ratio. We created four classification models by 5-fold cross-validation using the “train” function in the “caret” package. The importance of each gene calculated by the four methods was scored and ranked. Select the top 5 genes in terms of importance as the key for subsequent evaluation and validation of model diagnostic efficiency. At the same time, in order to determine the role of model genes in the process of gastritis-to-cancer transformation, we constructed diagnostic models for CG, LGIN, HGIN, and EGC based on each stage of evolution. And drew Venn plots by taking the intersection of the top five genes of importance in all stages of CG-LGIN-HGIN-EGC-GC to identify the core driving genes that stably play a role throughout the entire stage.

### Diagnostic model efficiency and validation

Use the “rms” and “ggDCA” packages to build the nomogram, calibration, and decision curve analysis (DCA) curves of the diagnostic model to test its diagnostic efficiency. In order to further evaluate the accuracy of the GC diagnostic model, we used the data from GSE79973 as the validation set to validate the GC diagnostic model and assess its accuracy and stability in prognosis.

### Enrichment analysis of core driver genes and protein-protein interaction (PPI) network

To further investigate the gene sets that were identified as key drivers in EGC samples using the aforementioned technique, we used Gene Set Enrichment Analysis (GSEA) [27] to examine the pathways and processes that might promote the emergence of GC. And through the STRING website (https://string-db.org/), perform interaction analysis between proteins enriched in pathways and driver gene proteins and construct an interaction network.

### Prognostic and immunological analysis of target gene CHI3L1

The high and low expression of the CHI3L1 gene on the prognosis and survival of GC patients were obtained from the Kaplan-Meier Plotter (https://kmplot.com/analysis/) database [28] and the BEST (https://rookieutopia.hiplot.com.cn/app_direct/BEST/) database [29], respectively. The single-cell resolution data analysis was conducted using the GSE167297 dataset [30] integrated from the Single cell and Spatially resolved Cancer Resources (SCAR) (http://scaratlas.com/) database [31]. Cell quality control was performed using the “Seurat” [32] standard with 200 < nFeatures < 6000 and MT < 10%. Normalize the gene expression matrix and identify highly variable genes using the ‘NormalizeData’ and ‘FindVariable Features’ functions, respectively, and perform principal component analysis (PCA) dimensionality reduction clustering and clustering. Observe the expression of CHI3L1 and CD44 in specific cell populations.

### Immunohistochemical staining

The human GC tissue microarrays were purchased from Shanghai Outdo Biotech, China. De-wax the tissue microarray in an oven at 63℃ for one hour. Perform antigen retrieval, anti-CHI3L1 antibody incubation, and secondary antibody incubation sequentially. Use the automated immunohistochemistry instrument with the specified program from the “Autostainer Link 48 Operating Instructions” Stain with hematoxylin for 1 min, mount with neutral balsam, and scan the images using the Aperio scanner (Aperio XT, LEICA). HistoScore = ((1×% weakly stained cells) + (2×% moderately stained cells) +(3×% strongly stained cells)).

The gastric mucosa of the rats was subjected to multiple immunohistochemical staining. Utilizing tyramide signal amplification (TSA) technology, the concurrent labeling of three distinct proteins within a single tissue section was accomplished. The procedure involves the following steps: dewaxing paraffin sections to water, performing antigen retrieval, blocking endogenous peroxidase activity with hydrogen peroxide, blocking non-specific binding with serum, applying the anti-α-SMA antibody (Servicebio, GB111364, ) and the HRP-labeled goat anti-rabbit antibody, applying the appropriate TSA reagent, conducting microwave treatment, repeating serum blocking, repeating the above steps and successively applying the anti-CD11c antibody (Servicebio, GB11059), and anti-CHI3L1 antibody (Proteintech, 12036-1-AP). The cell nuclei were stained with DAPI, autofluorescence was quenched, and the slides were mounted. Subsequently, the scanning was conducted utilizing a Pannoramic MIDI scanner (Hungary, 3DHISTECH).

### Rat model

Specific-pathogen-free (SPF) male Wistar rats, aged 4–5 weeks and weighing between 90 and 120 g, were procured from Sibef Biotechnology Co., LTD. (Beijing). The experimental procedures received approval from the Animal Research Ethics Committee of Beijing University of Chinese Medicine. Based on our team’s prior research on model generation, a quadrifactorial protocol incorporating 1-Methyl-3-nitro-1-nitrosoguanidine (MNNG) was utilized to induce a gastric mucosal carcinogenesis model in the rodent subjects [33]. The control group was supplied with standard SPF-grade chow and potable water, while the model group underwent a series of sequential interventions. Beginning in the first experimental week, the rodents in the model group were given ad libitum access to a 120 µg/mL MNNG potable solution and a diet of SPF-grade chow supplemented with 0.05% ranitidine, a histamine H2-receptor antagonist. In the following week, an irregular dietary regimen was introduced to simulate feast-famine cycles. In the third week, a 2% sodium salicylate solution was administered intragastrically.

### Cell model

Human GES-1 cells were procured from Wuhan Servicebio Technology Co., Ltd. and cultured in Dulbecco’s Modified Eagle Medium (DMEM; Thermo Fisher, USA) supplemented with 10% fetal bovine serum (FBS; Hyclone, USA), 100 U/mL penicillin, and 100 µg/mL streptomycin (Thermo Fisher). Human AGS cells were obtained from the Cell Bank / Stem Cell Bank of the Chinese Academy of Sciences and cultured in RPMI-1640 medium (Thermo Fisher) with 10% FBS (Hyclone, USA), 100 U/mL penicillin, and 100 µg/mL streptomycin (Thermo Fisher). MC cells were established as previously described [34]. In brief, the GES-1 cells were cultured to a density of 2 × 10^5^ cells/dish. During the logarithmic growth phase, the culture medium was replaced with DMEM complete medium containing 20 μm of MNNG. After 24 h of culture, the MNNG-containing medium was discarded, and the cells were then cultured in DMEM complete medium supplemented with 10% fetal bovine serum at 37℃ and 5% CO_2_. The GPL cells (MC) were obtained after approximately one week of culture.

### Cell Counting Kit-8 and wound healing assay

Seed cells into a 96-well plate and incubate overnight to allow for cell adhesion. Subsequently, based on prior research [35], 500 ng/mL of CHI3L1 was added to the culture medium, and the treatment was maintained for 72 h. Following this incubation period, add 10 µL of Cell Counting Kit-8 (CCK-8) solution to each well and return the plate to the incubator for an additional hour. Measure the optical density (OD) of each well at a wavelength of 450 nm using a microplate reader. Finally, calculate cell viability based on the obtained absorbance values.

Cells cultured in a 6-well plate were subjected to a scratch assay using a 10-µL pipette tip. Subsequently, 500 ng/mL of CHI3L1 was administered and incubated for 24 h at 37℃. Images were captured using a 4×objective lens on a microscope (Zeiss, Germany). The scratch area was quantitatively analyzed using Image J software (National Institutes of Health, USA), and the results were expressed as the percentage of wound closure. The percentage of wound closure was calculated using the formula: ((0 h area − 12/24 h area) / 0 h area)×100%.

### Western blot

The protein samples of tissues and cells were fractionated via SDS-PAGE and subsequently electrotransferred onto PVDF membranes. The membranes were initially incubated in TBST buffer supplemented with 5% non-fat milk to block non-specific binding sites, followed by incubation with the indicated primary antibody. Western blot analysis was performed using anti-CHI3L1 (Proteintech, 12036-1-AP), anti-CD44 (Proteintech, 60224-1-Ig), anti-β-catenin (BD, 610154), anti-c-Myc (Proteintech, 10828-1-AP), and anti-β-actin (Proteintech, 66009-1-Ig). Subsequently, the membranes were probed with the corresponding secondary antibody. Protein bands were detected using a chemiluminescent substrate (Bio-Rad) and quantified with an Imaging System (Qingxiang, China).

### RNA sequencing

The tissue lysates from rats were prepared using an RNA lysis buffer (Promega). The extracted RNA was then combined with purification beads to enhance its purity. Following successful quality control, the subsequent steps of library construction and sequencing were conducted by Shanghai OE Biotech Co., Ltd. (China). Subsequent data processing and analysis were conducted on the OE cloud platform.

### Human gastric mucosa derived organoid culture

The human gastric mucosa samples were obtained from patients who underwent gastroscopy examination at Dongfang Hospital of the Beijing University of Chinese Medicine (Beijing, China). Informed consent was obtained from all participants, and all procedures were conducted in compliance with the ethical standards and approval of the institutional review and ethics boards of Beijing University of Chinese Medicine (Approval Number: JDF-IRB-2023031702). The protocol for culturing organoids derived from human gastric mucosa was based on established methodologies as detailed in published literature [36]. The gastric mucosal tissue was precisely sectioned using a scalpel and subsequently incubated in 10 mL of chelation buffer solution containing 10 mM EDTA at 37 ℃ for 10–15 min. Post-incubation, the mixture was allowed to settle for one minute, and the supernatant was then discarded. Subsequently, 5 mL of pre-cooled Dulbecco’s Phosphate-Buffered Saline (DPBS) was added, and the tissue was gently pipetted up and down multiple times. The supernatant was collected and subjected to centrifugation at 4 ℃ and 300 g for 3 min to isolate the pellet. The gastric glands were then resuspended in 50 µL of matrix gel. This suspension was seeded into a 24-well plate and incubated in a cell culture incubator for 30 min prior to the addition of complete culture medium.

### Organoid Immunofluorescence staining

Organoids were seeded into Lab-Tek II 4-Well Sterile Chamber Slides and subsequently fixed with 3.7% formaldehyde at room temperature for 30 min. This was followed by three washes with phosphate-buffered saline (PBS). Permeabilization was achieved using 0.2% Triton X-100 for 15 min, after which the samples were blocked with PBS containing 1% bovine serum albumin (BSA) and 0.2% Triton X-100 for 30 min. Primary antibodies, including anti-β-catenin (BD, 610154), anti-phospho-β-catenin (CST, 9567 S), anti-CD44 (Proteintech, 60224-1-Ig), and anti-c-Myc (Proteintech, 10828-1-AP), were incubated overnight at 4 °C, followed by washes with PBS. Subsequently, secondary antibodies were incubated. The indicated antibody was incubated overnight at 4 ℃ again, followed by incubation of corresponding secondary antibody. Finally, DAPI was incubated at room temperature, and images were captured using a Nikon AX confocal microscope (Nikon, Japan).

### Data analysis

The bioinformatics analysis from the public databases was implemented based on R version 4.3.0. All experimental data were subjected to statistical analysis and visualized using GraphPad Prism 8 (GraphPad Software). Measurement data are presented as the mean ± standard error of the mean (SEM). The Student’s t-test compared two samples, while One-Way ANOVA compared multiple samples, followed by the Tukey test for significant results. Each experiment was independently replicated three times, and a p-value of less than 0.05 was considered to indicate statistical significance.

## Results

### Identification of biomarkers indicative of the progression from gastritis to cancer

**Fig. 1:**
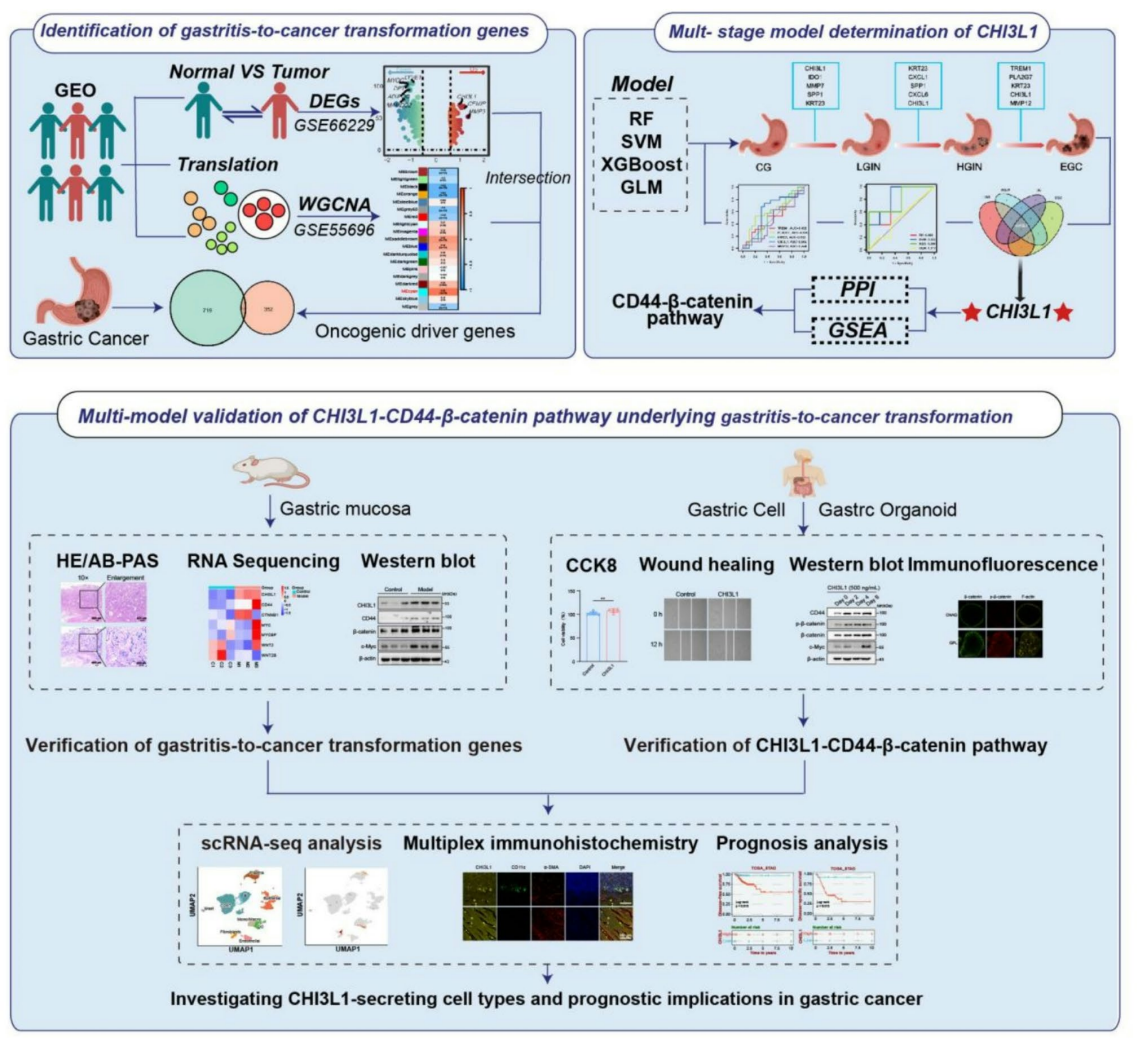
Flowchart illustrating the analysis and verification strategy

**Fig. 2:**
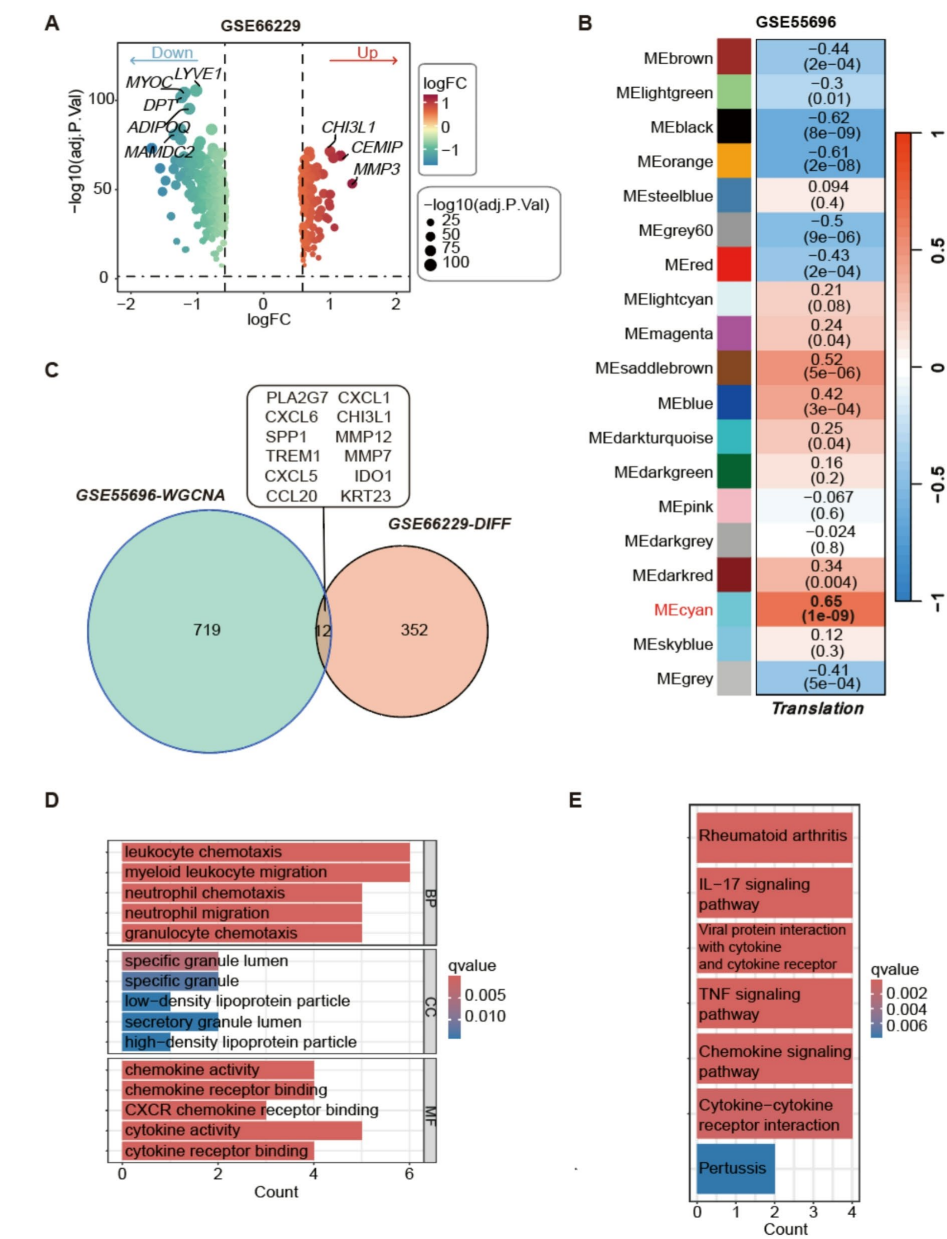
Identification of biomarkers associated with the progression from gastritis to cancer. (**A**) Volcanic map of differentially expressed genes in the GC dataset GSE66229. (**B**) Identification of transformation feature modules in the progression from gastritis to cancer within dataset GSE55696 utilizing WGCNA. (**C**) Based on the feature module of the gastritis to cancer transformation dataset and the intersection Venn diagram of differentially expressed genes in GC. (**D**) GO enrichment analysis of intersecting driving genes. (**E**) KEGG enrichment analysis of intersecting driving genes

The flowchart is shown in Fig. 1. Initially, we selected 364 differentially expressed genes from the GC dataset GSE66229 (Fig. 2A) to identify key genes involved in GC development. Concurrently, we employed the WGCNA method to analyze the GSE55696 dataset, which pertains to the transformation from gastritis-to-cancer, in order to extract gene clusters associated with transformation characteristics. A threshold of 180 was determined in accordance with the WGCNA method, and 7 outlier samples (GSM1341906, GSM1341900, GSM1341913, GSM1341907, GSM1341908, GSM1341905, and GSM1341914) were excluded from the analysis (Figure S1A). The optimal soft threshold is 12 (Figure S1B). Based on the similarity between modules, a total of 19 modules were obtained (Figure S1C-D). Among them, the cyan module gene showed the strongest correlation with the gastritis-to-cancer transformation (*R* = 0.65, *p* < 0.01, Fig. 2B). We intersected and overlapped genes related to the transformation of gastritis-to-cancer with differentially expressed genes in GC and ultimately identified 12 driver genes (*PLA2G7*, *CXCL1*, *CXCL6*, *CHI3L1*, *SPP1*, *MMP12*, *TREM1*, *MMP7*, *CXCL5*, *IDO1*, *CCL20*, *KRT23*) that ultimately lead to carcinogenesis (Fig. 2C). These genes are likely to play significant roles in the molecular mechanisms underlying gastritis-to-cancer transformation. GO and KEGG enrichment analyses revealed that 12 genes are predominantly associated with various immune cell chemotaxis responses, chemokine activity, and cancer-related pathways (Fig. 2D-E).

### Constructing a EGC diagnosis model through four types of machine learning

**Fig. 3:**
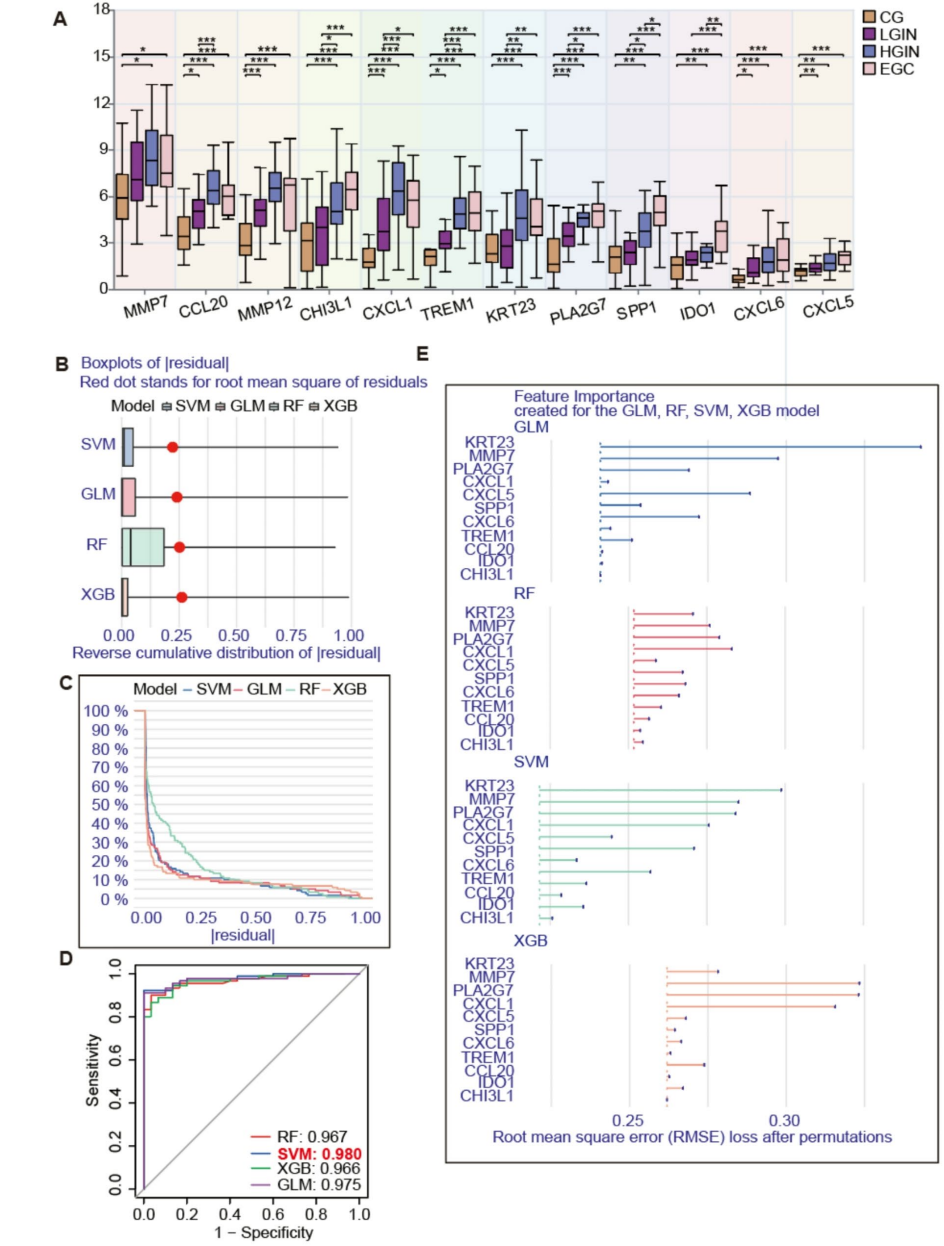
Constructing a EGC diagnosis model through four types of machine learning. (**A**) Comparison of expression levels of twelve driver genes during the progression from gastritis to GC. *p < 0.05, **p < 0.01, ***p < 0.001. (**B**) Residual plot of GC diagnostic model. (**C**) Reverse cumulative plot of GC diagnostic model. (**D**) Comparison curve of ROC accuracy for diagnostic models. (**E**) Comparative analysis of the significance of model genes across different diagnostic frameworks

We compared the expression levels of 12 driver genes in the GSE55696 dataset at four stages of gastritis-to-cancer transformation. Besides the *MMP7*, *CCL20*, *CXCL1*, and *KRT23* genes, the results showed that the mRNA expression levels of the other 8 genes generally went up as the disease got worse (Fig. 3A). To further screen genes that play a key role in GC diagnosis, we constructed a GC diagnosis model based on machine learning algorithms to identify 12 genes. We used four analysis methods, including RF, SVM, XGBoost, and GLM, among the 12 candidate genes. The findings indicated that the SVM method employed in the construction of the GC diagnostic model exhibited the lowest residual and reverse cumulative values (Fig. 3B-C), and the ROC curve demonstrated that the SVM diagnostic model had the highest accuracy (AUC = 0.98, Fig. 3D). Simultaneously, we evaluated and quantified the significance of the feature genes identified by the SVM model (Fig. 3E). Subsequently, we selected the top five genes with the highest importance scores for further investigation (*CHI3L1*, *MMP12*, *CXCL6*, *IDO1*, and *CCL20*).

### Validation of diagnostic models

**Fig. 4:**
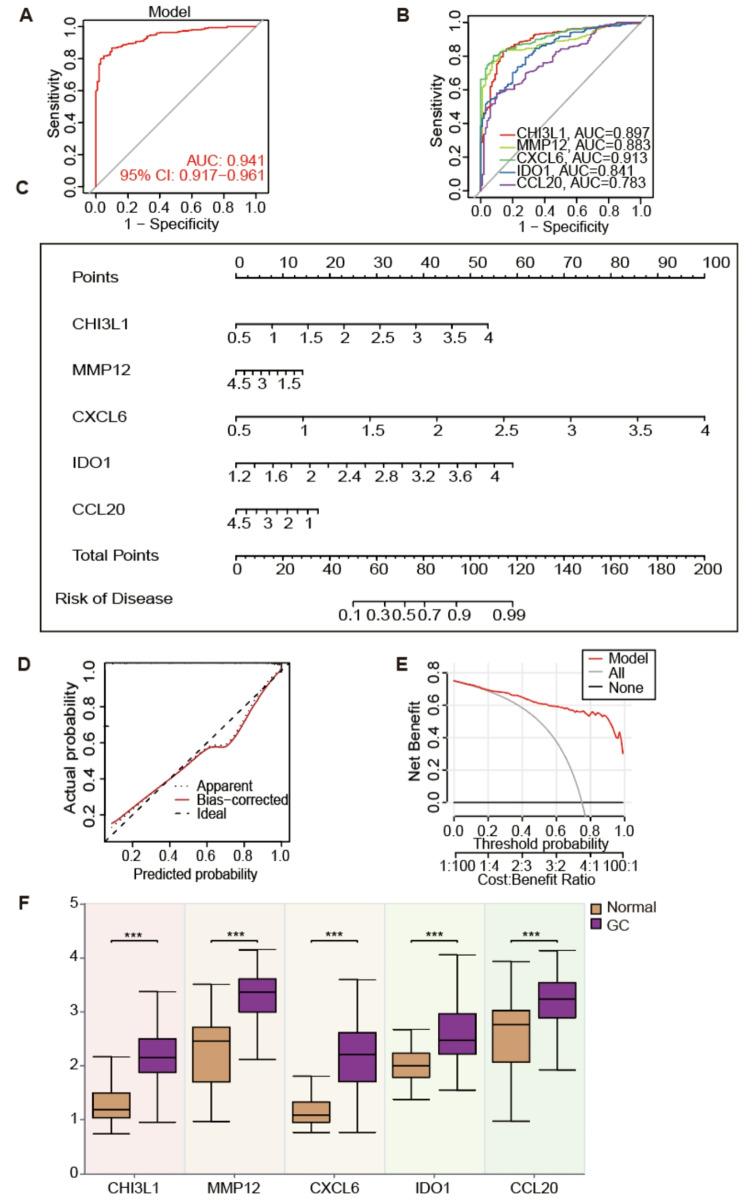
Validation of diagnostic models. (**A**) ROC curve illustrating the comprehensive diagnostic efficacy of the top five key genes within the model. (**B**) ROC plots illustrating the diagnostic efficacy of five key genes within the model. (**C**) A column chart constructed based on key genes. (**D**) Calibration curve of column chart. (**E**) DCA curve chart evaluates the accuracy and clinical benefits of column chart predictions. (**F**) Comparison of key genes in normal and GC tissues. ***p < 0.001

Based on the 5 key genes extracted earlier, we reevaluated the diagnostic efficacy, and the results showed that the accuracy of the GC diagnostic model was excellent (AUC = 0.941, Fig. 4A). The diagnostic efficacy of CXCL6 and CHI3L1 was excellent (AUC = 0.913, AUC = 0.896, Fig. 4B) in evaluating the value of 5 key genes. We used these 5 genes to construct a column chart to evaluate individual gene factors (Fig. 4C). The predicted values in the column chart and the actual observed values are pretty close to each other, as shown by the calibration curve. The DCA curve also shows that the model has good net clinical benefits (Fig. 4D-E).

To determine the expression levels of 5 genes in GC, we compared the mRNA expression differences between normal samples and GC tissue samples using the GSE66229 dataset. The results indicated that, relative to normal samples, the expression levels of 5 genes were significantly elevated in GC samples (Fig. 4F). Meanwhile, we evaluated the accuracy of the GC diagnostic model in the validation set GSE79973, and the ROC curve showed that the accuracy of the model diagnosis was relatively stable (AUC = 0.778, Figure S2A). The diagnostic performance of 5 key genes in the model showed that CHI3L1 and IDO1 had excellent diagnostic performance (AUC = 0.77, AUC = 0.73, Figure S2B). The nomogram constructed from key genes shows relatively stable predictive ability in the column chart (Figure S2C). At the same time, the calibration, based on the results from the nomogram and the DCA, demonstrates robust predictive performance. Furthermore, its application holds significant value for practical clinical decision-making (Figures S2D-E).

### Development of diagnostic models and identification of key targets in the progression from gastritis-to-cancer

Identifying the key driving genes that facilitate the progression from gastritis-to-cancer is crucial for elucidating the mechanisms underlying carcinogenesis. Consequently, employing the same methodology utilized for the construction of a GC diagnostic model, we developed a diagnostic model for each stage of the gastritis-to-cancer transformation continuum, specifically based on CG-LGIN-HGIN-EGC. And by observing the residuals and reverse accumulation plot, appropriate machine learning methods are determined to rank the importance of genes in the model, and ROC curves are used to evaluate the accuracy of the model and the role of each gene in the model. The ROC curve serves as a crucial instrument for assessing the performance of dichotomous models, particularly due to its capacity to directly illustrate the model’s generalization ability across varying classification thresholds. The Area Under the Curve (AUC) is quantified as the area beneath the ROC curve and functions as a metric for evaluating the learner’s performance. A higher AUC value indicates superior model performance.

The results showed that in the construction of the LGIN diagnostic model, the residual and reverse cumulative values of the SVM method were the lowest (Figures S3A-B), and the ROC curve demonstrated that the SVM diagnostic model had the highest accuracy (AUC = 0.72, Figure S3C-D). The top five genes *CHI3L1*, *IDO1*, *MMP7*, *SPP1*, and *KRT23* overall had good diagnostic accuracy in the model (AUC = 0.687, Figure S3E), among which the *IDO1*, *MMP7*, and *CHI3L1* genes had excellent diagnostic performance (AUC = 0.634, AUC = 0.619, AUC = 0.596; Figure S3F).

The XGBoost method has the lowest residual and reverse cumulative values according to the HGIN diagnostic model’s construction findings (Figures S4A-B). The SVM diagnosis model also exhibited the best accuracy, as seen by the ROC curve (AUC = 0.9, Figures S4C-D). In the model, the top five genes—*KRT23*, *CXCL1*, *SPP1*, *CXCL6*, and *CHI3L1*—had strong diagnostic accuracy (AUC = 0.821, Figures S4E). *CHI3L1* and *KRT23* genes, in particular, showed great diagnostic performance (AUC = 0.788, AUC = 0.734; Figure S4F).

**Fig. 5:**
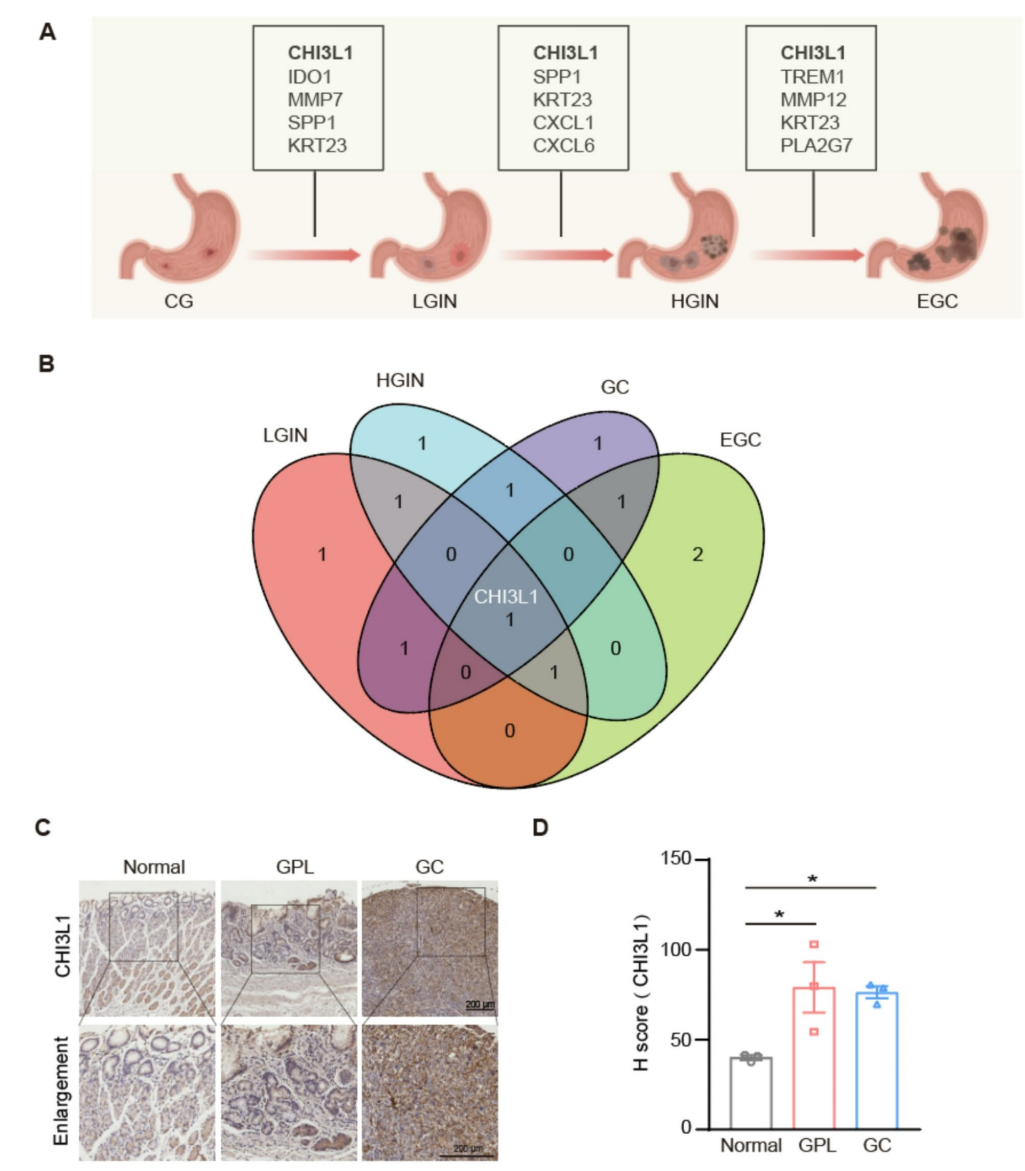
Development of diagnostic models and identification of key targets in the progression from gastritis-to-cancer. (**A**) Diagnostic biomarker profiles along the gastritis-to-cancer transformation. (**B**) The intersection of CG-LGIN-HGIN-EGC-GC diagnostic models at each stage generates the Venn map of the core driver gene. (**C**) Immunohistochemical analysis of CHI3L1 expression in human gastric tissue microarrays, including normal, GPL, and GC mucosa. (**D**) Quantitative analyses are described in (**C**). Data are given as the Average ± SEM. *p < 0.05

The SVM method demonstrated the lowest residual and reverse cumulative values (Figures S5A-B) and exhibited superior accuracy (AUC = 0.833, Figure S5C-D) in the construction of the EGC diagnostic model. In the model, the top five genes—*TMP12*, *PLA2G7*, *KRT23*, and *CHI3L1*—had high diagnostic accuracy (AUC = 0.718, Figure S5E). Among these genes, *CHI3L1* and *PLA2G7* had particularly strong diagnostic performance (AUC = 0.605, AUC = 0.605; Figure S5F). Figure 5 A shows the top 5 key genes from the diagnostic models of gastritis-to-cancer transformation. CHI3L1 was obtained as the core driver gene in the whole process of disease evolution (CG-LGIN-HGIN-EGC-GC) throughout the transformation (Fig. 5B).

To assess the significance of CHI3L1 in the progression from gastritis-to-cancer, we employed a human gastric mucosal tissue microarray to evaluate the expression levels of CHI3L1 across various pathological stages. As illustrated in Fig. 5C-D, both GPL and GC mucosa demonstrate elevated expression levels in comparison to normal mucosal tissue (*p* < 0.05).

### Enrichment analysis of *CHI3L1* gene, PPI network construction, and validation in rat model for gastric precancerous lesions

**Fig. 6:**
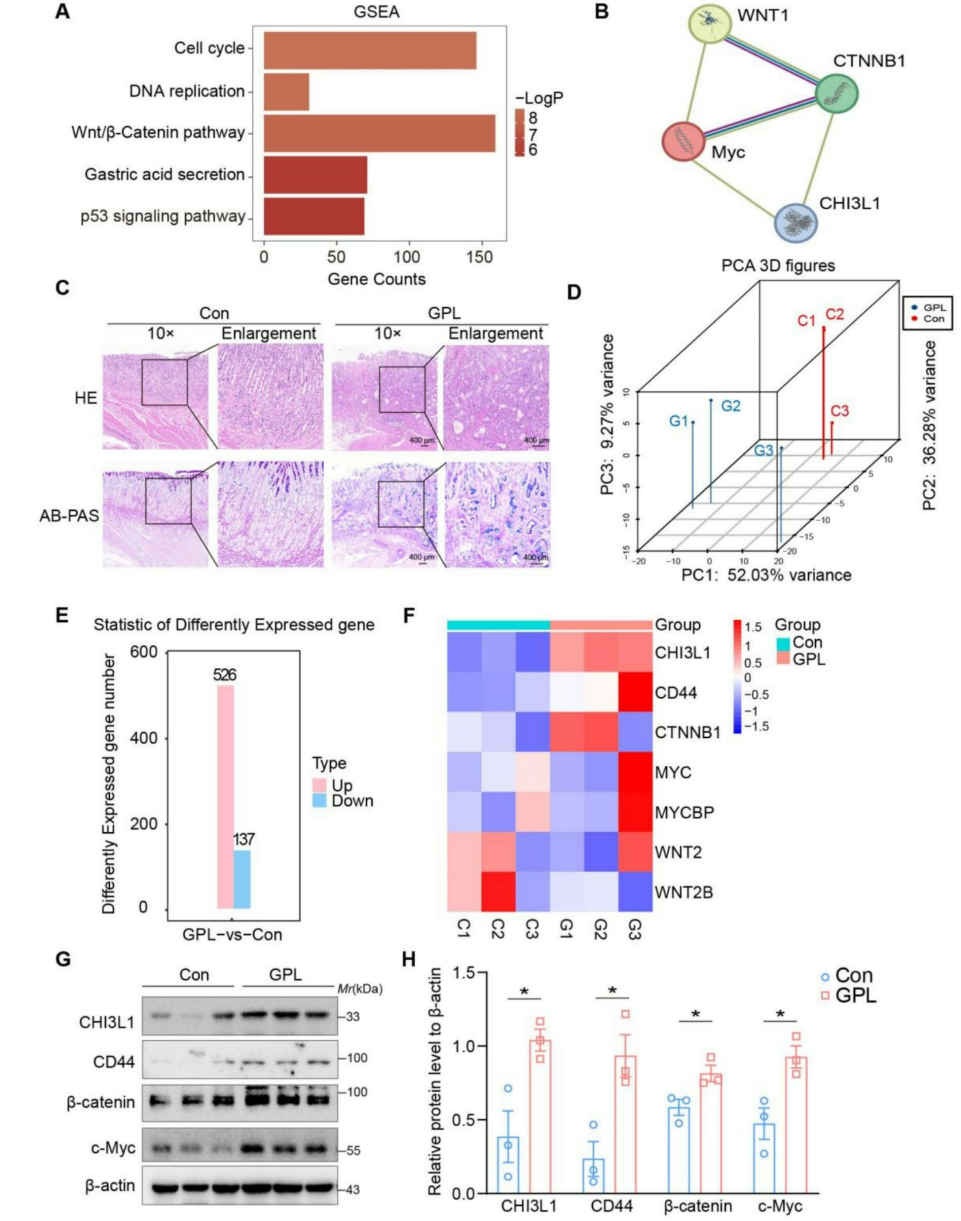
Enrichment analysis of *CHI3L1* gene, PPI network construction, and validation in rat model for gastric precancerous lesions. (**A**) Single gene GSEA enrichment analysis of CHI3L1. (**B**) PPI interaction network based on CHI3L1 protein and key proteins of Wnt/β-catenin pathway. (**C**) Hematoxylin and Eosin (HE) staining and Alcian Blue-Periodic Acid-Schiff (AB-PAS) staining of rat gastric mucosa (scale bar, 400 μm). (**D**) PCA was performed on transcriptome sequencing data derived from rat gastric mucosal tissue. (**E**) Histogram analysis of differential gene expression counts. (**F**) Heatmap analysis of CHI3L1, CD44, and Wnt/β-catenin pathway protein expression levels. (**G**) Western blot analysis of CHI3L1, CD44, β-catenin, and c-Myc protein levels. (**H**) The protein expression depicted in figure G was quantified relative to β-actin by utilizing integrated density values. All experiments were performed on three independent occasions, and data are given as the Average ± SEM. *p < 0.05

EGC development is influenced by the Wnt/β-Catenin, cell cycle, DNA replication, and stomach acid secretion pathways, as shown by the single gene GSEA enrichment study of *CHI3L1* in early carcinogenesis (Fig. 6A). The PPI analysis between the *CHI3L1* and pivotal genes within the Wnt/β-catenin signaling pathway revealed a direct interaction involving *CHI3L1*, *CTNNB1* (β-catenin), and *MYC*(Fig. 6B). These findings indicate that *CH13L1* potentially facilitate the development of GC by modulating β-catenin and its downstream effector *MYC* within the Wnt/β-catenin signaling pathway. To further substantiate the role of the core gene *CHI3L1* and associated pathway proteins, we developed a rat model of GPL, as illustrated in Figure S6A. Histological examination using Hematoxylin and Eosin (HE) staining and Alcian Blue-Periodic Acid-Schiff (AB-PAS) staining confirmed that the pathological characteristics of the rat gastric mucosa were consistent with those observed in GPL. In comparison to the control group, the gastric mucosa of the GPL rats exhibited distorted and crowded glands, characterized by cellular atypia, including enlarged, hyperchromatic nuclei, an elevated nuclear-cytoplasmic ratio, and a loss of cellular polarity(Fig. 6C). Subsequently, transcriptome sequencing was conducted on rat gastric mucosal tissue (Fig. 6D-E), followed by the validation of key genes that had been previously identified and ranked among the top 5 for diagnostic models at the gastritis-to-cancer transformation process. The results demonstrated that *CHI3L1* exhibited the most significant differential expression (Figure S6B).

According to prior literature, CD44 serves as the receptor for CHI3L1 and is implicated in the progression of GC [35]. Consequently, we employed a heatmap to analyze the expression levels of CHI3L1, CD44, and proteins associated with the Wnt/β-catenin signaling pathway. The findings are presented in Fig. 6F. Additional validation of these findings was performed utilizing Western blot analysis, which demonstrated a markedly elevated expression of CHI3L1, CD44, β-catenin, and c-Myc in GPL rats (Fig. 6G-H).

### CHI3L1 activates the CD44-β-catenin pathway, promoting malignancy in GPL cells

**Fig. 7:**
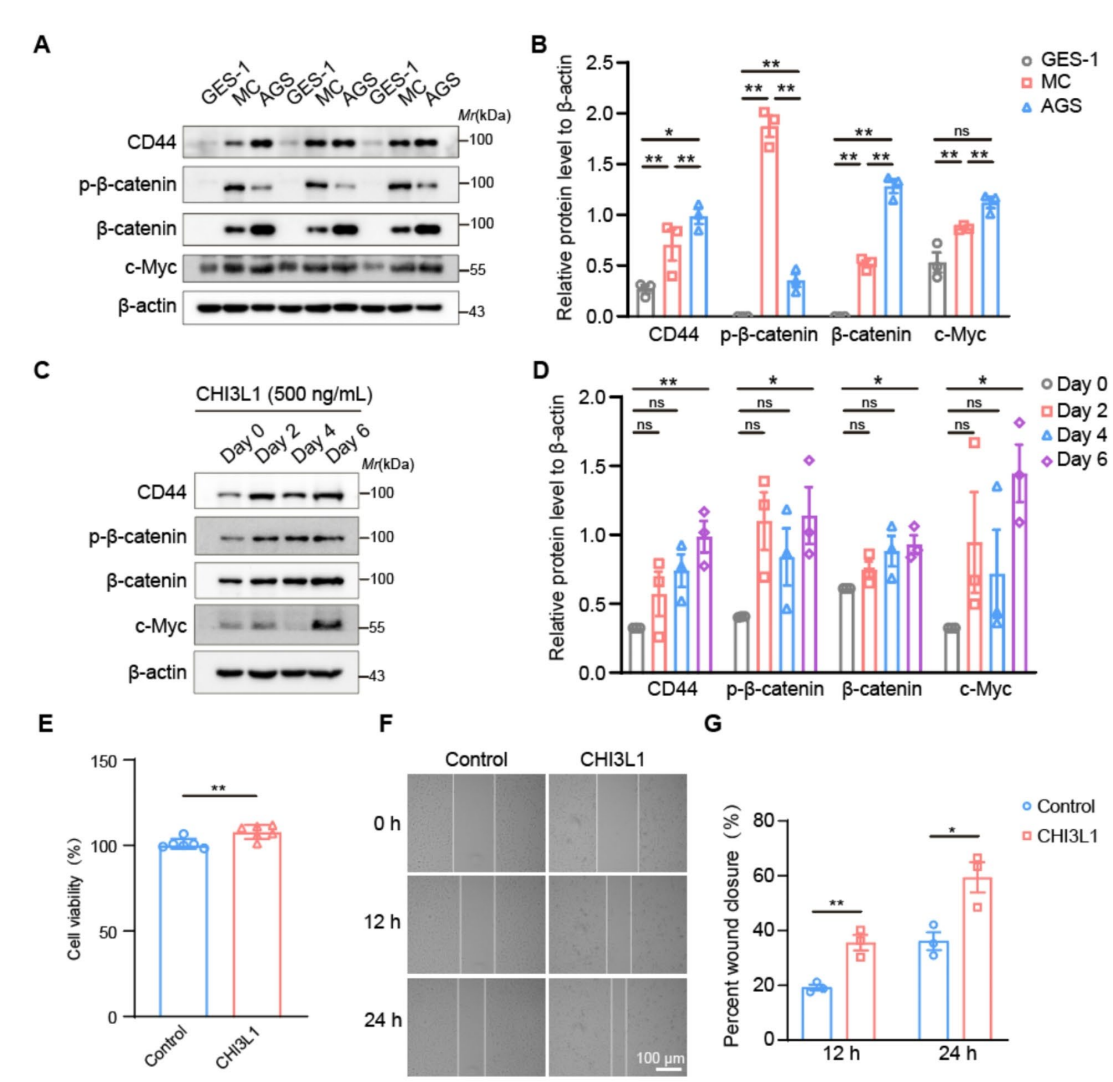
CHI3L1 activates the CD44-β-catenin pathway, promoting malignancy in GPL cells. (**A**) Western blot analysis of CD44, β-catenin, p-β-catenin, and c-Myc protein levels in GES-1, MC, and AGS cell lines. (**B**) Quantitative analyses are described in (A), and data are given as the Average ± SEM. ^ns^*p*>0.05, *p < 0.05, **p < 0.01. (**C**) Western blot analysis of CD44, β-catenin, p-β-catenin, and c-Myc in MC cells treated with CHI3L1 (500 ng/mL) for 0, 2, 4, and 6 days. (**D**) Quantitative analyses are described in (C). Data are given as the Average ± SEM. ^ns^*p*>0.05, *p < 0.05. (**E**) CCK8 analysis of cell viability after CHI3L1 (500 ng/mL) treatment for 72 h. Data are given as the Average ± SEM, **p < 0.01. (**F**) Wound healing assay of MC cells with or without CHI3L1 (500 ng/mL) stimulation (scale bar, 100 μm). (**G**) Quantitative analyses of the wound healing assay are described in (F). All experiments were performed on three independent occasions, and data are given as the Average ± SEM, *p < 0.05, **p < 0.01

To elucidate the underlying molecular mechanisms by which CHI3L1 influences the modulation of CD44 and β-catenin in the carcinogenic process, we initially generated a cell line model for GPL, termed MC cells, in accordance with the methodology previously reported [34]. Western blot analysis was employed to assess the protein expression levels of CD44, β-catenin, phosphorylated β-catenin (p-β-catenin), and c-Myc in human gastric epithelial GES-1 cells, MC cells, and AGS cells. The results indicated that the expression levels of CD44, β-catenin, p-β-catenin, and c-Myc were significantly elevated in both MC and AGS cells compared to GES-1 cells (Fig. 7A-B).

Subsequently, we supplemented the MC cell culture medium with 500 ng/mL of CHI3L1 and collected cell samples at various time points for western blot analysis. The findings indicated an upregulation in the protein levels of CD44, β-catenin, p-β-catenin, and c-Myc commencing from the second day of CHI3L1 treatment (Fig. 7C-D). To further substantiate the impact of CHI3L1 stimulation on the malignant phenotype of the cells, we employed the CCK8 assay to assess cell viability after CHI3L1 treatment. The findings demonstrated that a 48-hour treatment with CHI3L1 at a concentration of 500 ng/mL significantly enhanced cellular proliferation (*p* < 0.01, Fig. 7E). Furthermore, in wound healing assay, CHI3L1 was observed to increase cellular migration capabilities (*p* < 0.01, Fig. 7F-G).

### The CD44-β-catenin-c-Myc signaling pathway is highly active in GPL organoids

**Fig. 8:**
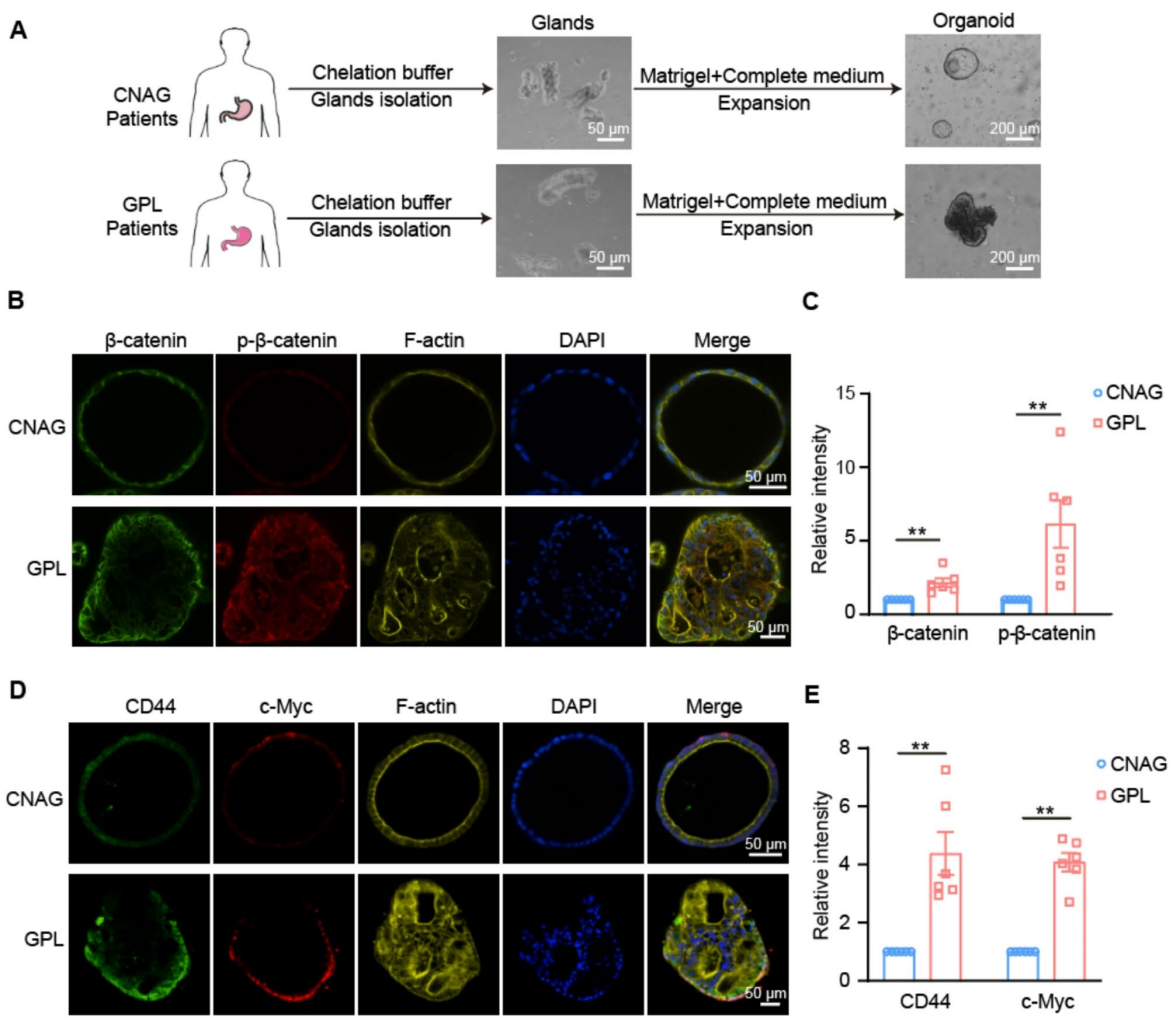
The CD44-β-catenin-c-Myc signaling pathway is highly active in GPL organoids. (**A**) Human gastric mucosa derived organoid construction process and morphology of isolated glands and organoids under the light microscope. (**B**) Immunofluorescence staining of β-catenin, and p-β-catenin in cultured organoids (scale bar, 50 μm). (**C**) Quantitative analyses are described in (B), data are given as the Average ± SEM. **p < 0.01. (**D**) Immunofluorescence staining of CD44, and c-Myc in cultured organoids (scale bar, 50 μm). (**E**) Quantitative analyses are described in (D). All experiments were performed on three independent occasions, and data are given as the Average ± SEM, **p < 0.01

To further verify the activation of CD44-β-catenin-c-Myc signaling pathway in GPL,

gastric mucosal samples were collected from patients undergoing gastroscopy, and organoids were subsequently constructed from the gastric mucosa of individuals diagnosed with chronic non-atrophic gastritis (CNAG) and GPL. The comprehensive procedure for the establishment of organoids is thoroughly documented in the literature [36]. Figure 8A shows the construction process and morphology of isolated glands and organoids under the light microscope. Organoids derived from the gastric mucosa of CNAG patients demonstrated well-polarized cellular cavities and spheroid structures. In contrast, organoids derived from GPL exhibited solid structures, a loss of cellular polarity, and irregularly layered formations. Immunofluorescence staining of cultured organoids revealed that, in comparison to organoids derived from CNAG, there was a marked upregulation in the fluorescence intensity of CD44, β-catenin, p-β-catenin, and c-Myc protein expression in organoids from GPL (*p* < 0.01, Fig. 8B-E). These findings further corroborate the pronounced activation of the CD44-β-catenin-c-Myc signaling pathway in GPL.

### CHI3L1, mainly secreted by fibroblasts and dendritic cells, is linked to poor prognosis in gastric cancer

**Fig. 9:**
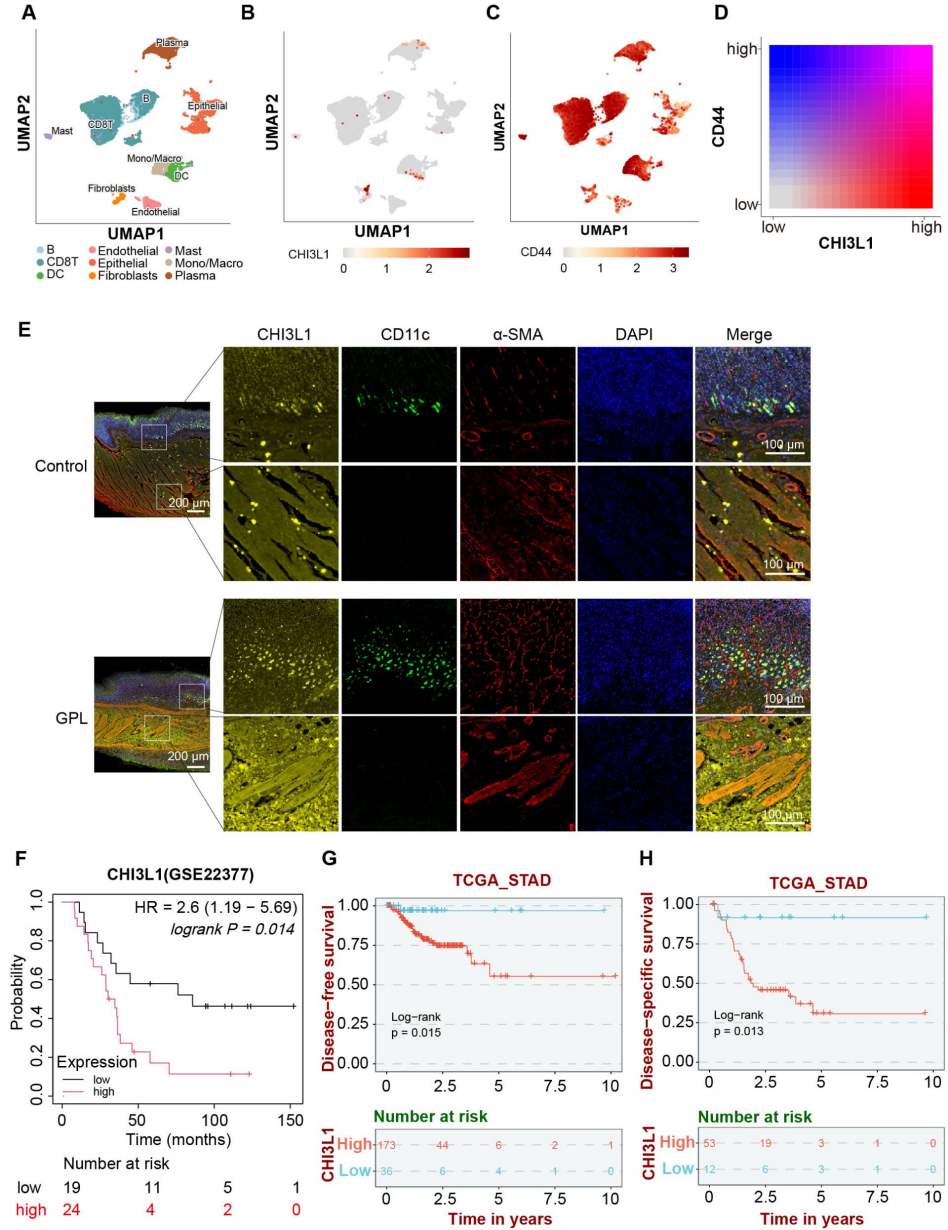
CHI3L1, mainly secreted by fibroblasts and dendritic cells, is linked to poor prognosis in gastric cancer. (**A**) Cell subpopulation clustering results of GSE167297 dataset. (**B**) The expression distribution of CHI3L1 at single-cell resolution. (**C**) The expression distribution of CD44 at single-cell resolution. (**D**) The co expression results of CHI3L1 and CD44 in single cells. (**E**) Co-immunofluorescence staining for α-SMA, CD11c, and CHI3L1 in control and GPL rat gastric mucosa (scale bar, 100 μm). (**F**) Differences in prognostic outcomes among patients with high and low expression of CHIL31 in the GSE22377 dataset. (**G**) DFS survival outcomes of patients with high and low CHIL31 expression in the TCGA database. (**H**) The DSS survival outcomes of patients with high and low CHIL31 expression in the TCGA database

Gene expression profiling at the single-cell resolution is instrumental in elucidating cell type-specific gene expression patterns. To analyze the cell types responsible for CHI3L1 secretion, we obtained 9 cell subpopulations based on the GSE167297 dataset (Fig. 9A). CHI3L1 was mainly concentrated in fibroblasts and dendritic cells (Fig. 9B), while CD44 was abundantly expressed in various cell subpopulations, with the highest expression levels in CD8 + T cells, dendritic cells, and Macro/Mono cells (Fig. 9C). The results of single gene coexpression showed a positive correlation between the expression levels of CHI3L1 and CD44 at single-cell resolution (Fig. 9D).

To verify that CHI3L1 is predominantly secreted by fibroblasts and dendritic cells, we performed multiple immunohistochemical analyses on rat gastric mucosa. We employed anti-α-SMA antibody to label fibroblasts, anti-CD11c antibody to identify dendritic cells, and concurrently stained for CHI3L1 to examine the distribution of fibroblasts, dendritic cells, and CHI3L1 within the gastric mucosa. The findings revealed that fibroblasts were ubiquitously distributed across the mucosal layer, dendritic cells were predominantly localized in the lamina propria of the mucosa, and CHI3L1 expression was markedly observed throughout the entire mucosal layer (Fig. 9E).

To elucidate the prognostic implications of varying CHI3L1 expression levels in GC patients, we analyzed survival outcomes using the GSE22377 dataset available from the Kaplan-Meier Plotter database. The Kaplan-Meier curve of survival results showed that the overall survival rate (OS) of patients with low CHI3L1 expression was higher than that of patients with high expression (*p* < 0.05, Fig. 9F). Supplementary analysis was conducted on the Disease − free survival (DFS) and Disease − specific survival (DSS) results of GC patients obtained from the BEST database and TCGA database. The patients exhibiting low CHI3L1 expression demonstrated superior DFS and DSS outcomes compared to those with high CHI3L1 expression (*p* < 0.05, Fig. 9G-H).

## Discussion

In recent years, despite a decline in the incidence of GC, the prevalence of the disease remains considerable, contributing significantly to the global disease burden. Consequently, the pursuit of early prediction and diagnosis of GC continues to be a critical strategy for reducing its incidence. GC, a common digestive malignancy, evolves from gastritis to carcinoma, with GPL being the last stage and independent risk factors for GC development [37, 38]. Studying this phase can reveal the mechanisms behind this transformation and help identify early intervention targets for GC [39]. Our study integrated machine learning techniques with dataset analyses pertaining to GC and gastritis-to-cancer transformation, ultimately identifying CHI3L1 as the pivotal driver gene throughout the gastritis-to-cancer continuum. Subsequently, we conducted an in-depth analysis of the CHI3L1-related pathway and verified its downstream signaling, and associated malignant cell phenotypes. Furthermore, we characterized the cell types responsible for CHI3L1 secretion and found CHI3L1 is associated with poor prognosis in GC.

The progression from chronic gastritis to GC adheres to a multi-step, multi-factorial cascade reaction model [40, 41]. As this cascade progresses, the risk of developing GC incrementally increases. Strategies of primary prevention focus on improving the detection and eradication of the *Helicobacter pylori* [42]. Although the widespread use of endoscopic screening enables the early detection of GC, its invasive nature limits its broader adoption and application [43]. Numerous researchers are investigating biomarkers that could potentially replace or complement gastroscopy, with the objective of facilitating the early detection of GC [39, 44, 45]. Our study identified 12 driver genes (*PLA2G7*, *CXCL1*, *CXCL6*, *CHI3L1*, *SPP1*, *MMP12*, *TREM1*, *MMP7*, *CXCL5*, *IDO1*, *CCL20*, *KRT23*) that contribute to carcinogenesis during the progression of chronic gastritis-to-cancer transformation. Additionally, we developed an EGC diagnostic model utilizing 4 types of machine learning algorithms. Among these models, the SVM method demonstrated the lowest residual and reverse cumulative values, as well as the highest accuracy. Notably, the genes of *CHI3L1*, *MMP12*, *CXCL6*, *IDO1*, and *CCL20* exhibited the highest importance scores in the model. This indicates their potential utility in constructing a more precise early diagnostic model for GC, thereby facilitating the timely detection of the disease in its initial stages.

CHI3L1, also referred to as YKL-40, belongs to the evolutionarily conserved 18-glycosyl-hydrolase protein family and is expressed in a diverse array of cell types, including endothelial cells, macrophages, neutrophils, chondrocytes, fibroblasts, and epithelial cells [46, 47]. CHI3L1 is intricately associated with the onset and progression of various inflammatory diseases and inflammation related tumors [48, 49]. In the context of GC, CHI3L1 is upregulated and positively correlated with the depth of tumor invasion, lymph node status, and tumor staging [50, 51]. CHI3L1 interacts with its receptor CD44, leading to the activation of the AKT and ERK pathways and the phosphorylation of β-catenin. This interaction promotes malignant phenotypes [35]. CD44, a single-pass transmembrane glycoprotein, is expressed on embryonic stem cells, connective tissue, and bone marrow cells. Elevated expression of CD44 has been observed in individuals with gastric lesions that have progressed along the gastric precancerous cascade, as well as in those with *Helicobacter pylori*-positive gastritis [52, 53]. CD44 is upregulated in malignant cells and acts as a marker for gastric stem cells. Activated by CHI3L1, CD44 affects the cell cycle, proliferation, differentiation, EMT, and metabolism via Wnt/β-catenin, PI3K/AKT, and ERK signaling pathways, which are crucial for GC progression and metastasis [54–56].

Among these pathways, activation of the Wnt/β-catenin pathway leads to the accumulation of β-catenin in the nucleus, promoting the expression of tumorigenesis-related genes such as c-Myc, which is closely associated with the development of GC [57]. The PI3K/AKT pathway regulates cell growth, metabolism, and survival, and is more closely linked to the progression of gastric cancer [58]. Activation of the ERK pathway is typically triggered by the overexpression or mutation of growth factor receptors (e.g., EGFR), contributing to enhanced cell proliferation and survival [59]. In this study, we concentrate on the GPL stage and demonstrate that CHI3L1 expression is significantly elevated in both GPL and GC tissues. The significant release of CHI3L1 effectively regulates the CD44-β-catenin-c-Myc signaling pathway in both in vitro and in vivo models, which contributes to the promotion of malignant cellular phenotypes. These findings elucidate a potential mechanism underlying the transformation from gastritis-to-cancer, thereby establishing a theoretical foundation for the development of targeted therapeutic agents aimed at this pathway. Naturally, there remains considerable scope for the enhancement and optimization of core gene acquisition algorithms based on machine learning [60]. This includes refining the SVM algorithm [61] for more effective gene selection, improving overall model performance, and integrating multiple novel model combinations to collaboratively construct more robust systems [62, 63]. Such advancements will be instrumental in facilitating the identification and screening of more suitable potential targets.

Given the significant role of CHI3L1 in the progression from gastritis-to-cancer, we conducted an analysis to identify the cell types responsible for its secretion. Our findings indicate that CHI3L1 is predominantly secreted by fibroblasts and dendritic cells, and its expression correlates with poor prognosis in GC. This is consistent with previous research that integrated proteomics and transcriptome analysis, identifying plasma CHI3L1 as a potential biomarker for patients with endoscopically resectable GC [14]. Therefore, assessing the expression levels of CHI3L1 can facilitate the identification of GPL patients at elevated risk for progression to GC, while also serving as a valuable reference for clinicians in the development of treatment strategies. Future research should focus on creating precise CHI3L1-targeted liquid biopsy platforms using advanced methods to measure CHI3L1 in various biofluids [64]. Integrating these biomarkers with clinical and histopathological data via machine learning techniques may lead to the development of a robust diagnostic system, thereby improving the early detection of EGC and facilitating timely interventions for high-risk populations.

## Conclusion

This study highlights CHI3L1 as a key gene in the transition from gastritis to cancer, confirming its role in promoting cancerous behavior through the CD44-β-catenin-c-Myc pathway. Measuring CHI3L1 levels in serum or body fluids could improve early detection and intervention in EGC. Inhibiting the secretion levels of CHI3L1 or targeting the blockade of its downstream signaling pathways may represent a potentially effective strategy for preventing the malignant progression, thus warranting further investigation and validation.

## Electronic supplementary material

Below is the link to the electronic supplementary material.

Supplementary Material 1

Supplementary Material 2

## Data Availability

The data achieved and analyzed in the current study are available in the GEO database (https://www.ncbi.nlm.nih.gov/geo/); further reasonable inquiries can be directed to the corresponding author.

## Abbreviations

GC: Gastric cancer
EMT: Epithelial-mesenchymal transition
GPL: Gastric precancerous lesions
GEO: Gene Expression Omnibus
CG: Chronic gastritis
LGIN: Low-grade intraepithelial neoplasia
HGIN: High-grade intraepithelial neoplasia
EGC: Early gastric cancer
WGCNA: Weighted Gene Co-Expression Network Analysis
TOM: Topological overlap matrix
GS: Gene significance
MM: module membership
DGE: Differential gene expression
GO: Gene Ontology
KEEG: Kyoto Encyclopedia of Genes and Genomes
RF: Random forest
SVM: Support vector machine
DCA: Decision curve analysis
PPI: Protein-protein interaction
TSA: Tyramide signal amplification
SPF: Specific-pathogen-free
MNNG: 1-Methyl-3-nitro-1-nitrosoguanidine
CCK8: Cell Counting Kit-8
ROC: Receiver operating characteristic
AB-PAS: Alcian Blue-Periodic Acid-Schiff
CNAG: Chronic non-atrophic gastritis
OS: Overall survival rate
DFS: Disease − free survival
DSS: Disease − specific survival
