# Supplementary material for: CHI3L1: a key driver in gastritis-to-cancer transformation
Source: J Transl Med. 2025 Mar 19;23:349. doi: 10.1186/s12967-025-06352-2 (PMC11921547; doi:10.1186/s12967-025-06352-2)
Supplement: Supplementary file 1 — Supplementary Material 1 [file 12967_2025_6352_MOESM1_ESM.docx]

Supplementary Materials for

**CHI3L1: A Key Driver in Gastritis-to-Cancer Transformation**

**Tao Li^†1^, Huizhong Jiang^†2^, Yucheng Gong^†2^, Mengting Liao^2^, Yuanping Jia^2^, Jiena Chen^2^, Ming Dai^3^, Yinan Yan^1^, Xinyu Lu^2^, Runhua Chen^4^, Yuan Li^5,6^, Yan Chen^1^, Jie Lin^5,6^, Yicong Li^4^*, Xia Ding^1,2,6^***

**^1^School of Traditional Chinese Medicine, Beijing University of Chinese Medicine, Beijing 100029, China**

**^2^Dongzhimen Hospital, Beijing University of Chinese Medicine, Beijing, 100700, China**

**^3^MOE Key Laboratory of Membraneless Organelle and Cellular Dynamics, Hefei National Laboratory for Physical Sciences at the Microscale, University of Science and Technology of China, Hefei, 230027, China**

**^4^Dongfang Hospital, Beijing University of Chinese Medicine, Beijing, 100078, China**

**^5^National Institute of Traditional Chinese Medicine Constitution and Preventive Treatment of Diseases, Beijing University of Chinese Medicine, Beijing, 100029, China**

**^6^Research Center for Spleen and Stomach Diseases of Traditional Chinese Medicine, Beijing University of Chinese Medicine, Beijing, 100029, China**

**^†^These authors made equal contributions to this work.**

**Correspondence to:**

**liyicong330@163.com (Yicong Li)**

**[dingx@bucm.edu.cn](mailto:Dingxia@bucm.edu.cn) (Xia Ding)**

**This file includes:**

Figures. S1 to S6

Fig S1


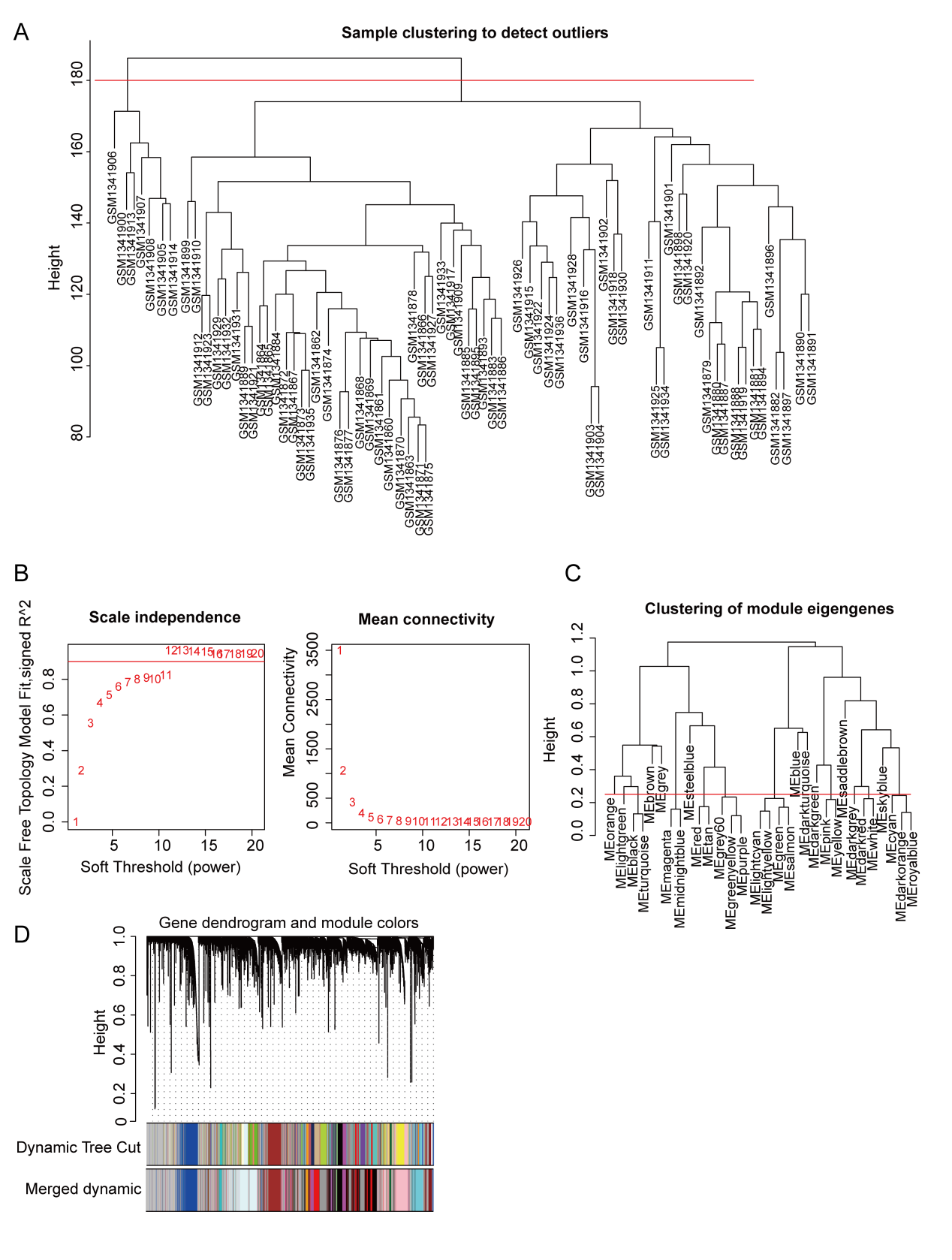


**Fig S1. WGCNA identifies the disease-relevance modules.**

(**A**) Results of sample cluster tree filtering. (**B**) Screening using a soft threshold. (**C**) Cutting and merging of the module cluster tree based on the specified threshold. (**D**) Results of module merging.

Fig S2


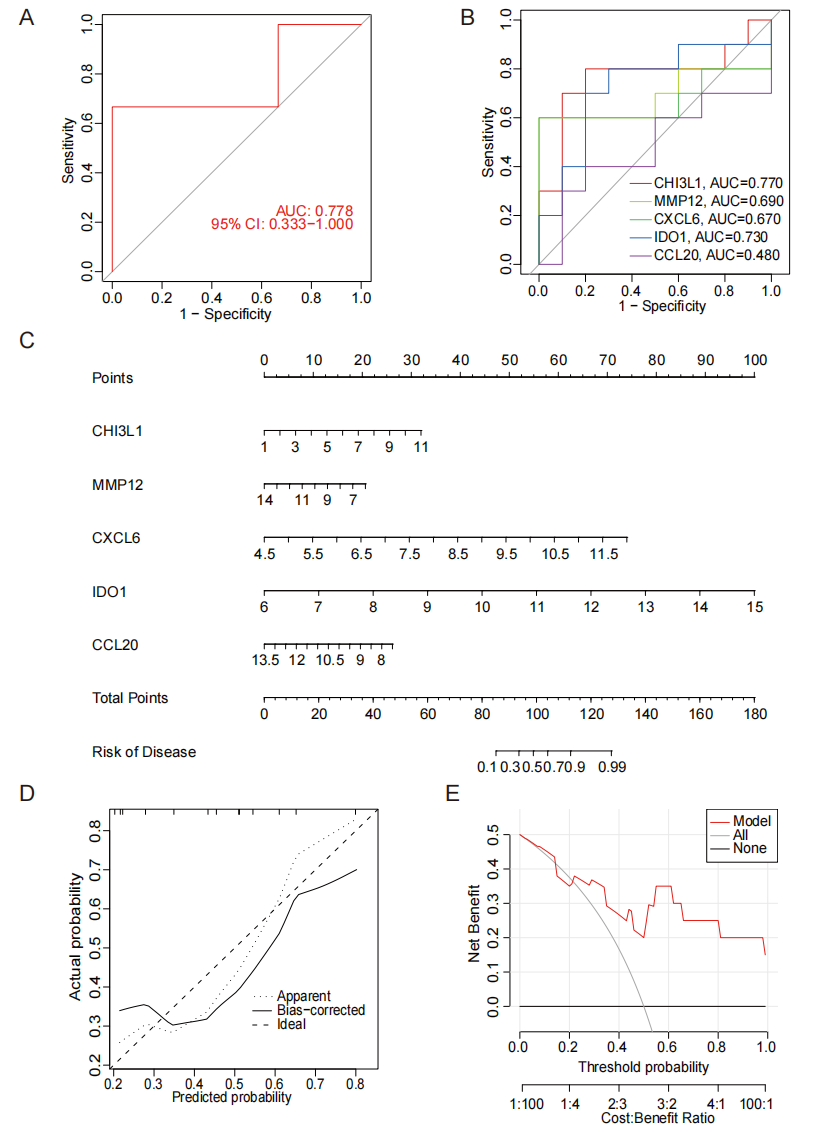


**Fig S2. Validation of diagnostic model of GSE79973 dataset.**

(**A**) ROC plot of the overall diagnostic performance of the top five key genes in the model. (**B**) ROC plots of the diagnostic value of 5 key genes in the model. (**C**) A nomogram constructed based on key genes. (**D**) Calibration curve of nomogram. (**E**) DCA curve chart used to test the accuracy of nomogram prediction and clinical benefits. (**F**) Comparison of key genes in normal and gastric cancer tissues.

Fig S3


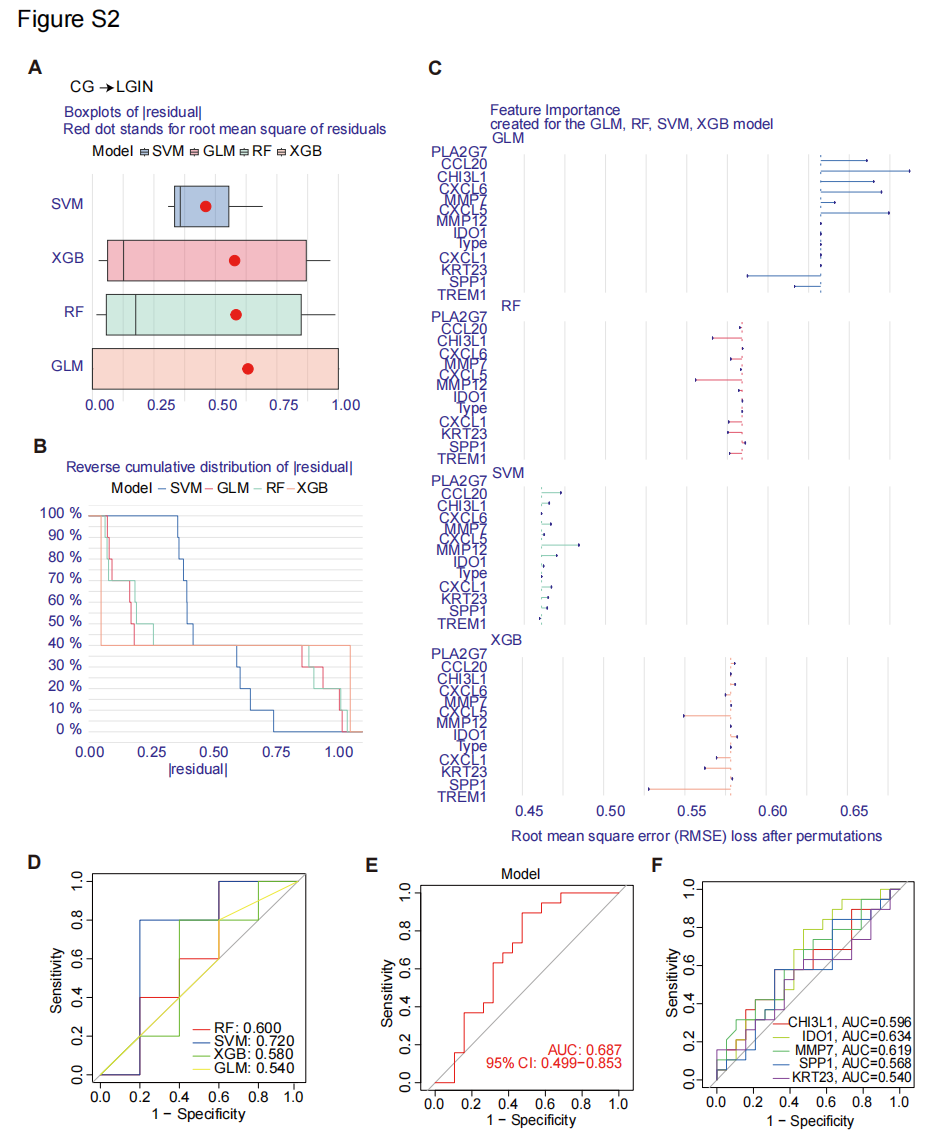


**Fig S3. Four machine learning methods for constructing LGIN diagnostic models.**

(**A**) Residual plot of LGIN diagnostic model. (**B**) Reverse cumulative plot of LGIN diagnostic model. (**C**) Comparison of the importance of model genes in various diagnostic models. (**D**) ROC accuracy comparison curve of diagnostic models. (**E**) ROC plot of the overall diagnostic performance of the top five key genes in the model. (**F**) ROC plots of the diagnostic value of 5 key genes in the model.

Fig S4


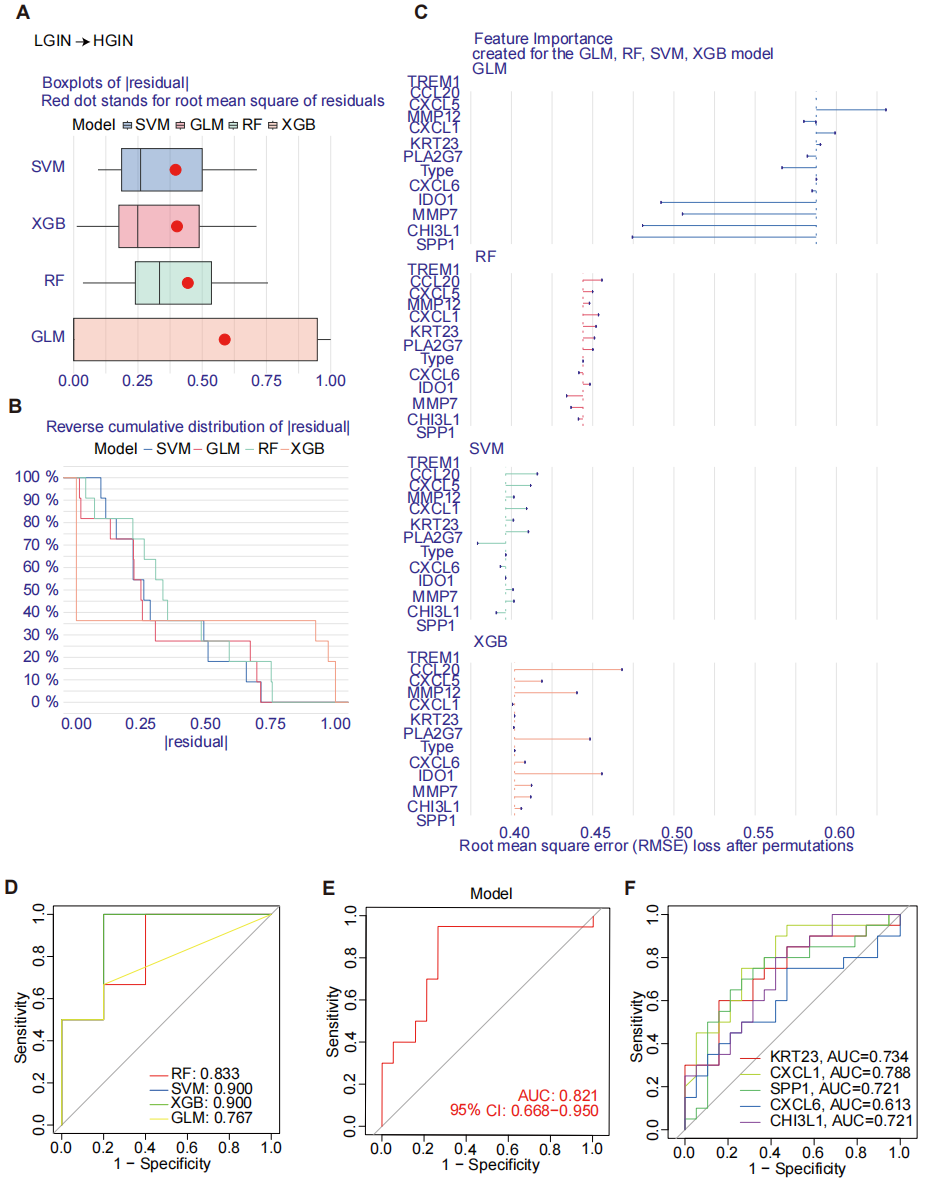


**Fig S4. Four machine learning methods for constructing HGIN diagnostic models.**

(**A**) Residual plot of HGIN diagnostic model. (**B**) Reverse cumulative plot of HGIN diagnostic model. (**C**) Comparison of the importance of model genes in various diagnostic models. (**D**) ROC accuracy comparison curve of diagnostic models. (**E**) ROC plot of the overall diagnostic performance of the top five key genes in the model. (**F**) ROC plots of the diagnostic value of 5 key genes in the model.

Fig S5


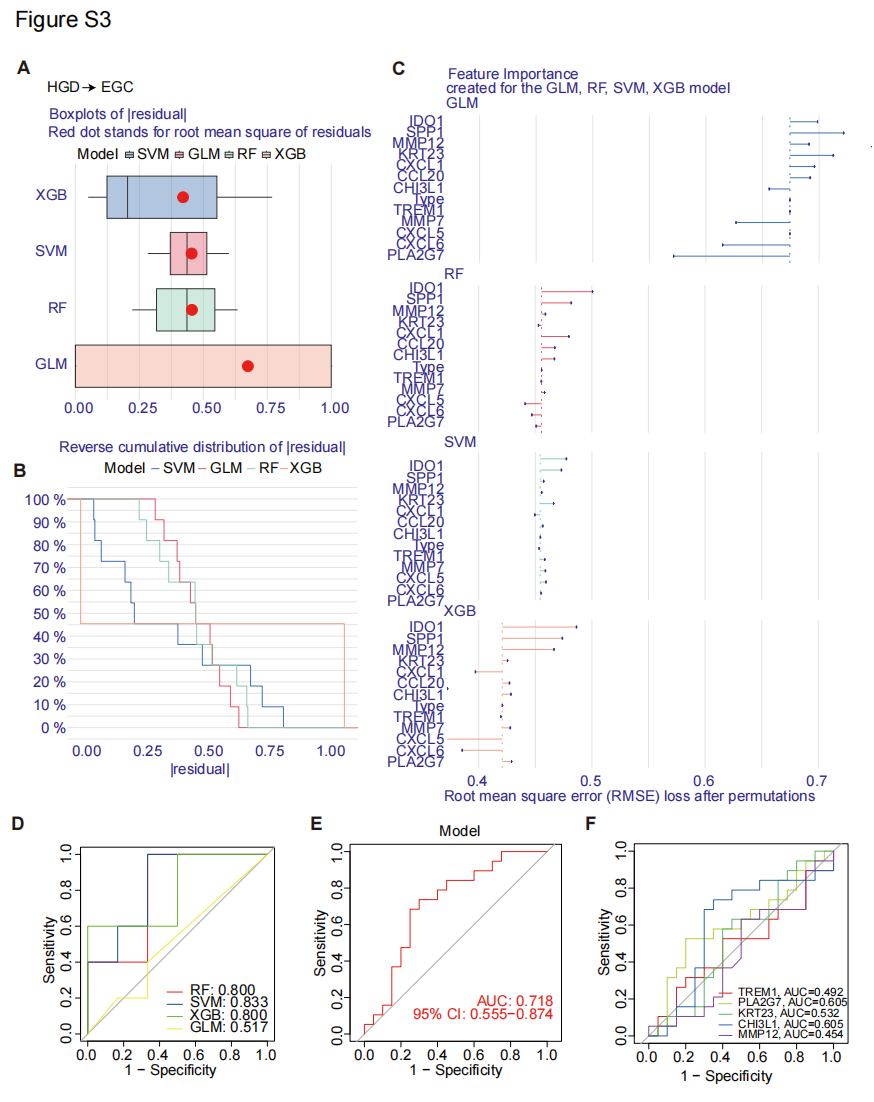


**Fig S5. Four machine learning methods for constructing EGC diagnostic models.**

(**A**) Residual plot of EGC diagnostic model. (**B**) Reverse cumulative plot of EGC diagnostic model. (**C**) Comparison of the importance of model genes in various diagnostic models. (**D**) ROC accuracy comparison curve of diagnostic models. (**E**) ROC plot of the overall diagnostic performance of the top five key genes in the model. (**F**) ROC plots of the diagnostic value of 5 key genes in the model.

Fig S6


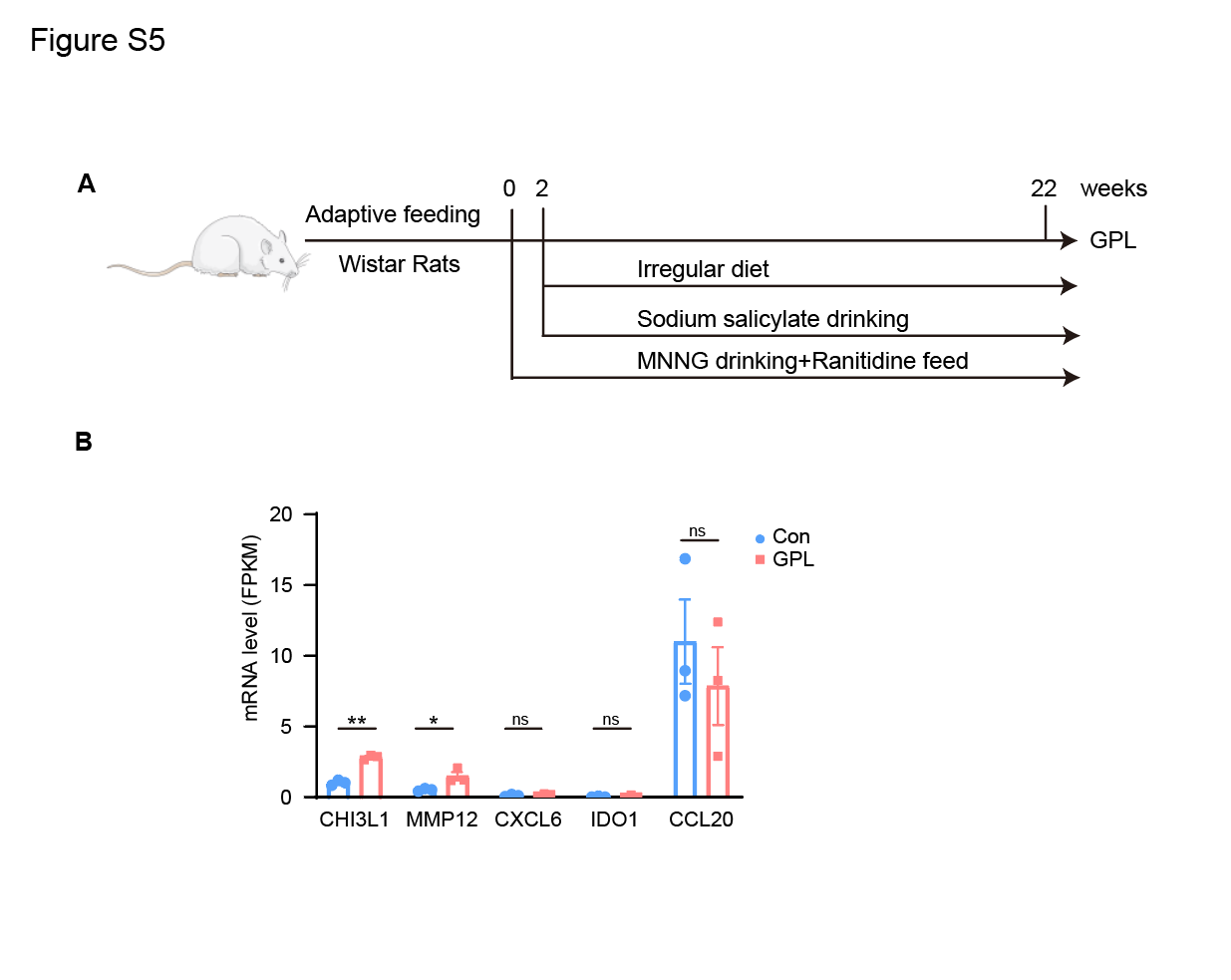


**Fig S6. Flowchart for creating a GPL rat model and validating the top five key diagnostic genes at each stage of gastritis-to-cancer progression.**

(**A**) Flowchart for constructing a GPL rat model. (**B**) Validation of top five key genes previously identified for diagnostic models at each stage of the gastritis-to-cancer transformation.
