# Supplementary material for: CHI3L1: a key driver in gastritis-to-cancer transformation
Source: J Transl Med. 2025 Mar 19;23:349. doi: 10.1186/s12967-025-06352-2 (PMC11921547; doi:10.1186/s12967-025-06352-2)
Supplement: Supplementary file 2 — Supplementary Material 2 [file 12967_2025_6352_MOESM2_ESM.pdf]

Figure S1

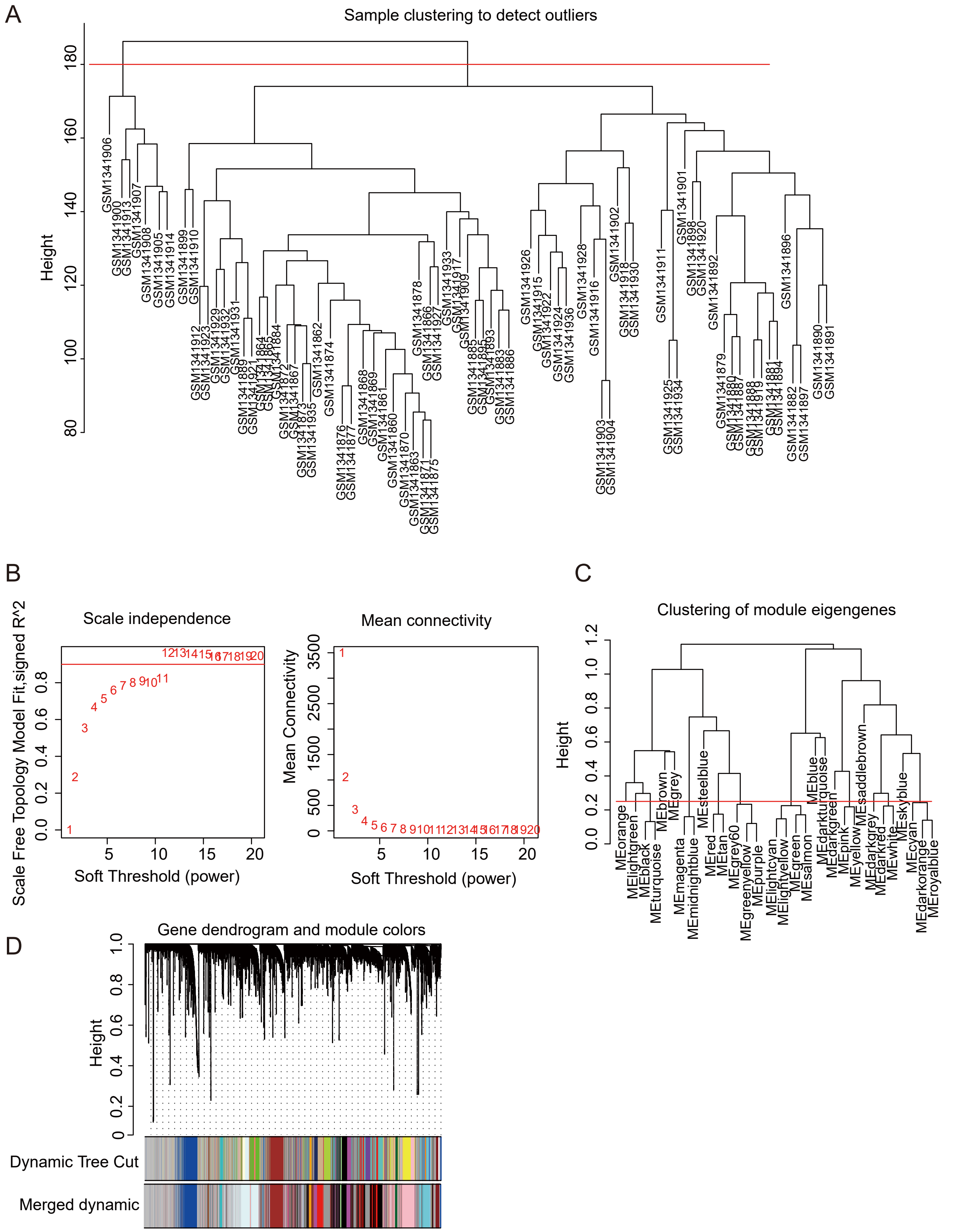

A
Sample clustering to detect outliers
Height
B
Scale independence
Scale Free Topology Model Fit,signed R^2
Soft Threshold (power)
Mean connectivity
Mean Connectivity
Soft Threshold (power)
C
Clustering of module eigengenes
Height
D
Gene dendrogram and module colors
Height
Dynamic Tree Cut
Merged dynamic

Figure S2

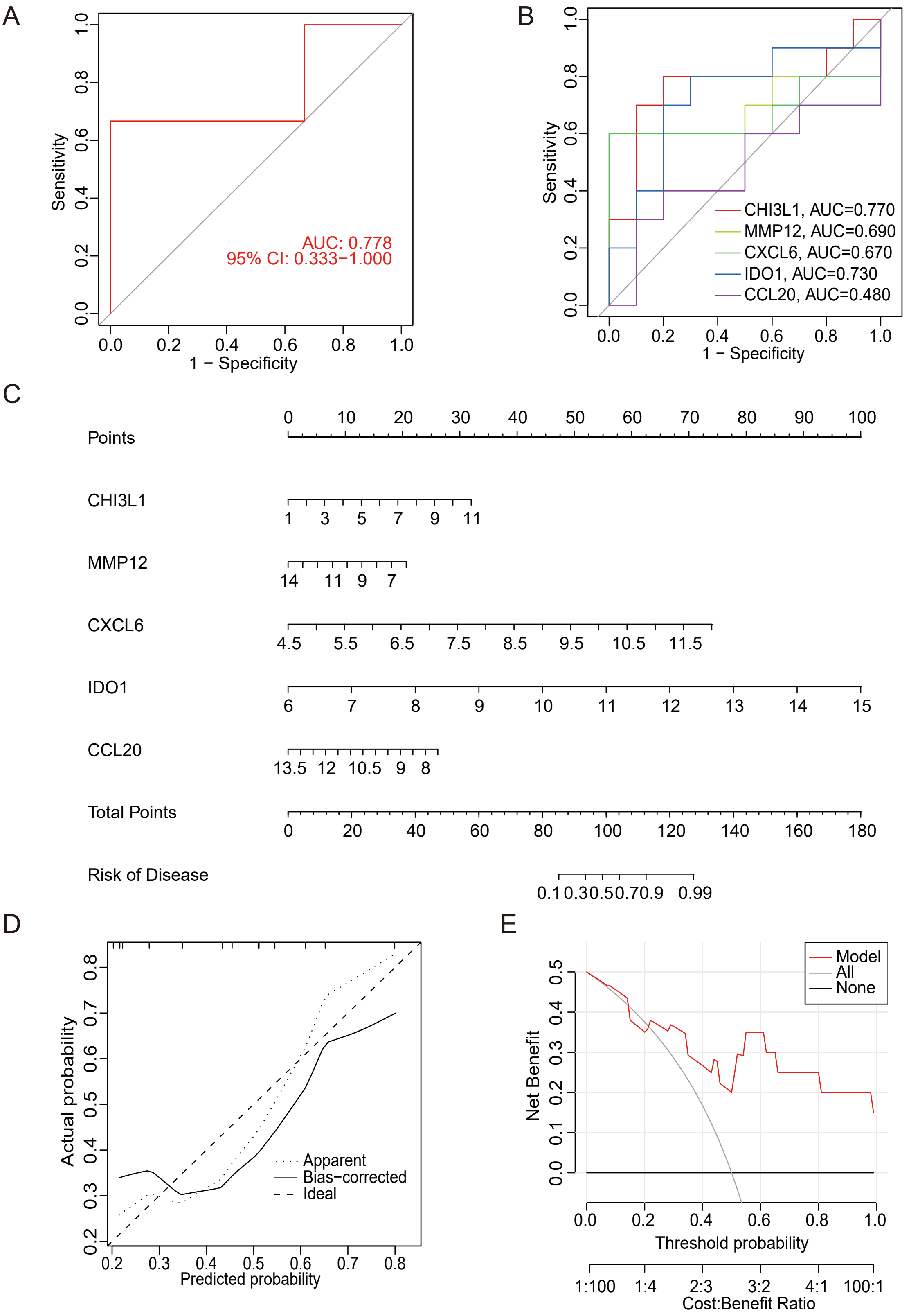
A
Sensitivity
1 − Specificity
AUC: 0.778
95% CI: 0.333−1.000
B
CHI3L1, AUC=0.770
MMP12, AUC=0.690
CXCL6, AUC=0.670
IDO1, AUC=0.730
CCL20, AUC=0.480
C
Points
CHI3L1
MMP12
CXCL6
IDO1
CCL20
Total Points
Risk of Disease
0.1 0.3 0.5 0.7 0.9 0.99
D
Actual probability
Predicted probability
Apparent
Bias−corrected
Ideal
E
Net Benefit
Threshold probability
Model
All
None
1:100 1:4 2:3 3:2 4:1 100:1
Cost:Benefit Ratio

Figure S3

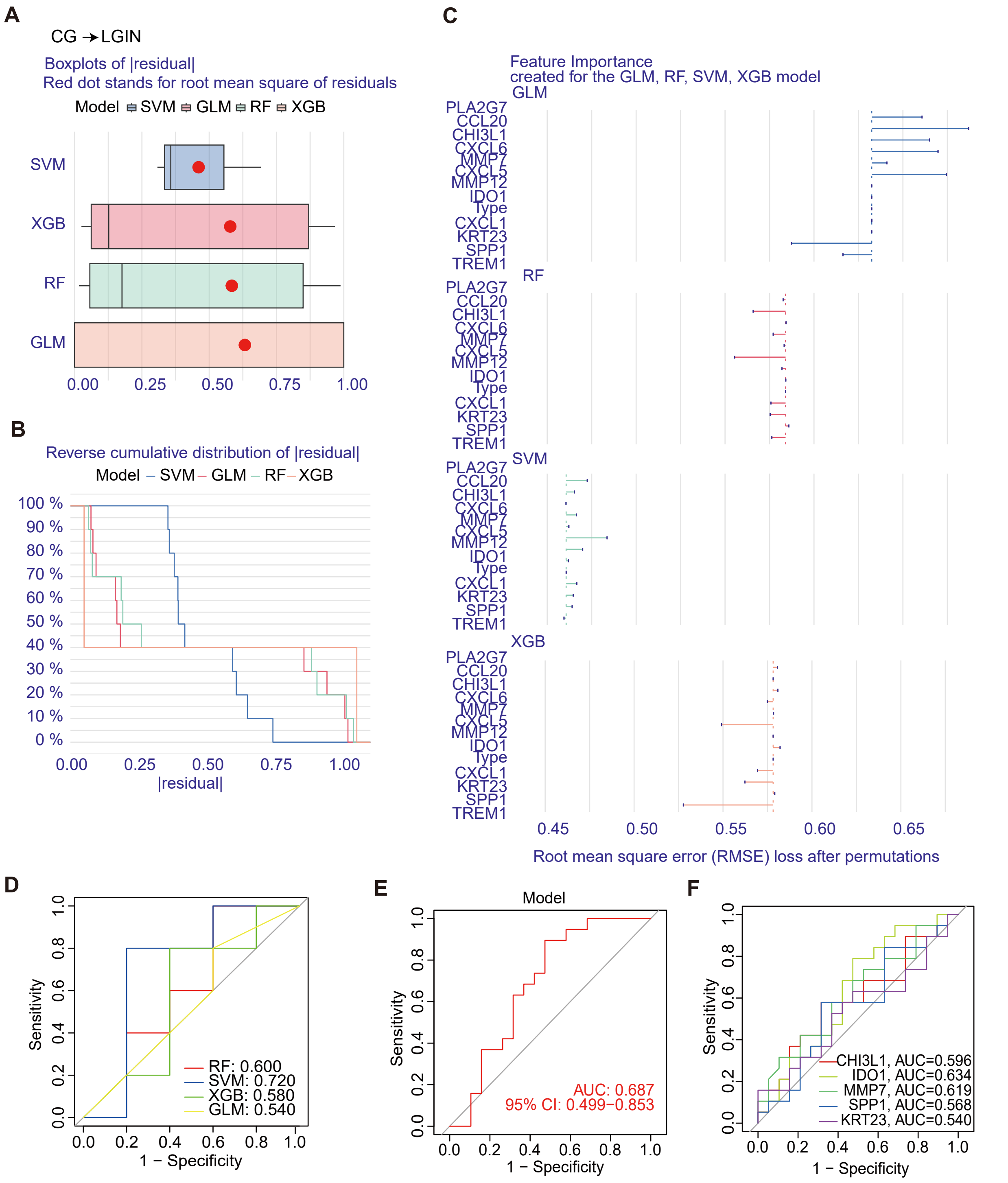

A
CG → LGIN
Boxplots of |residual|
Red dot stands for root mean square of residuals
Model SVM GLM RF XGB
SVM
XGB
RF
GLM
0.00 0.25 0.50 0.75 1.00
B
Reverse cumulative distribution of |residual|
Model SVM GLM RF XGB
100 % 90 % 80 % 70 % 60 % 50 % 40 % 30 % 20 % 10 % 0 %
0.00 0.25 0.50 0.75 1.00
|residual|
C
Feature Importance
created for the GLM, RF, SVM, XGB model
GLM
RF
SVM
XGB
PLA2G7
CCL20
CHI3L1
CXCL6
MMP7
CXCL5
MMP12
IDO1
Type
CXCL1
KRT23
SPP1
TREM1
0.45 0.50 0.55 0.60 0.65
Root mean square error (RMSE) loss after permutations
D
Sensitivity
1 − Specificity
RF: 0.600
SVM: 0.720
XGB: 0.580
GLM: 0.540
E
Model
Sensitivity
1 − Specificity
AUC: 0.687
95% CI: 0.499−0.853
F
Sensitivity
1 − Specificity
CHI3L1, AUC=0.596
IDO1, AUC=0.634
MMP7, AUC=0.619
SPP1, AUC=0.568
KRT23, AUC=0.540

Figure S4

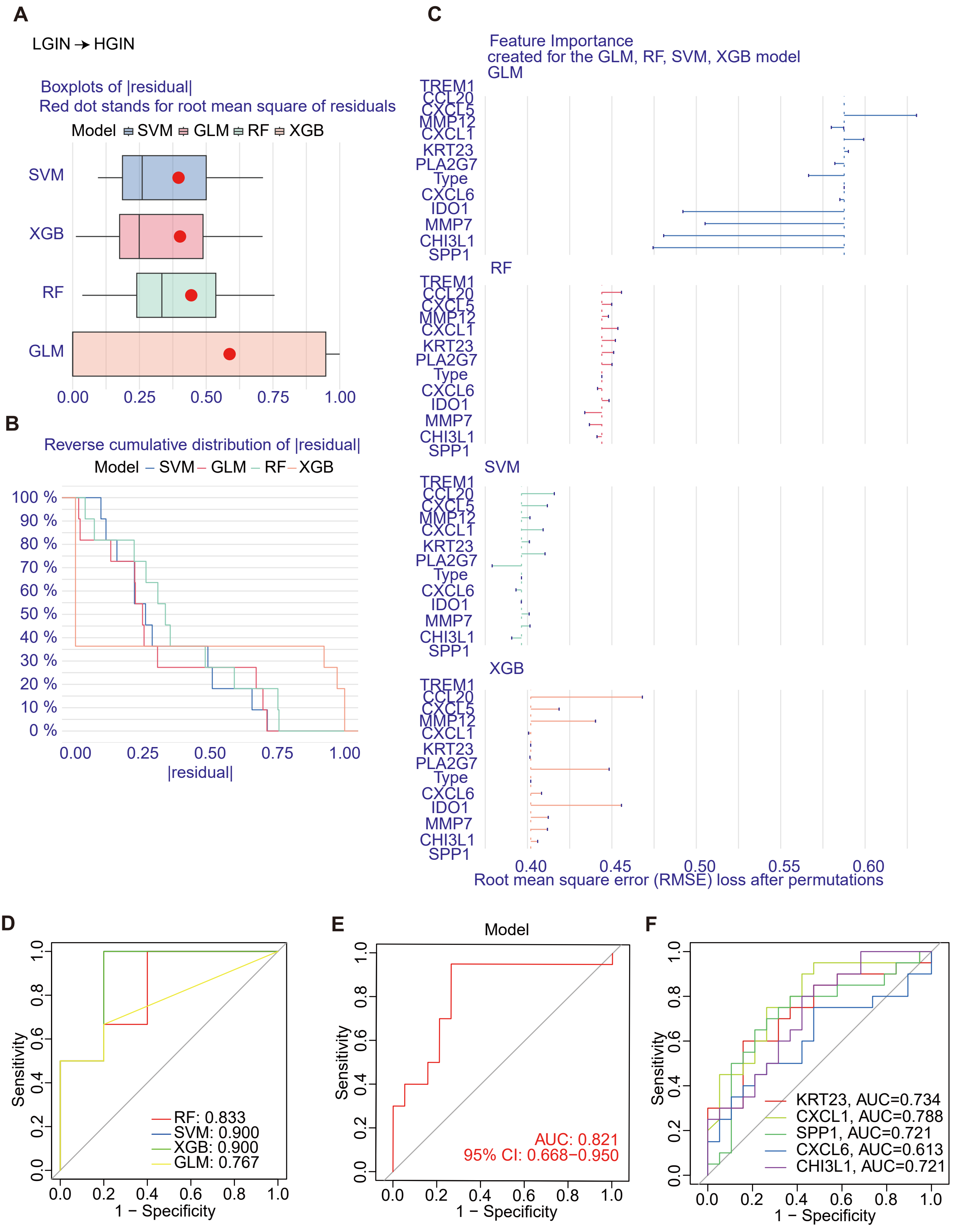
A
LGIN → HGIN
Boxplots of |residual|
Red dot stands for root mean square of residuals
Model SVM GLM RF XGB
SVM
XGB
RF
GLM
0.00 0.25 0.50 0.75 1.00
B
Reverse cumulative distribution of |residual|
Model SVM GLM RF XGB
100 % 90 % 80 % 70 % 60 % 50 % 40 % 30 % 20 % 10 % 0 %
0.00 0.25 0.50 0.75 1.00
|residual|
C
Feature Importance
created for the GLM, RF, SVM, XGB model
GLM
RF
SVM
XGB
TREM1
CCL20
CXCL5
MMP12
CXCL1
KRT23
PLA2G7
Type
CXCL6
IDO1
MMP7
CHI3L1
SPP1
0.40 0.45 0.50 0.55 0.60
Root mean square error (RMSE) loss after permutations
D
Sensitivity
1 − Specificity
RF: 0.833
SVM: 0.900
XGB: 0.900
GLM: 0.767
E
Model
Sensitivity
1 − Specificity
AUC: 0.821
95% CI: 0.668−0.950
F
Sensitivity
1 − Specificity
KRT23, AUC=0.734
CXCL1, AUC=0.788
SPP1, AUC=0.721
CXCL6, AUC=0.613
CHI3L1, AUC=0.721

Figure S5

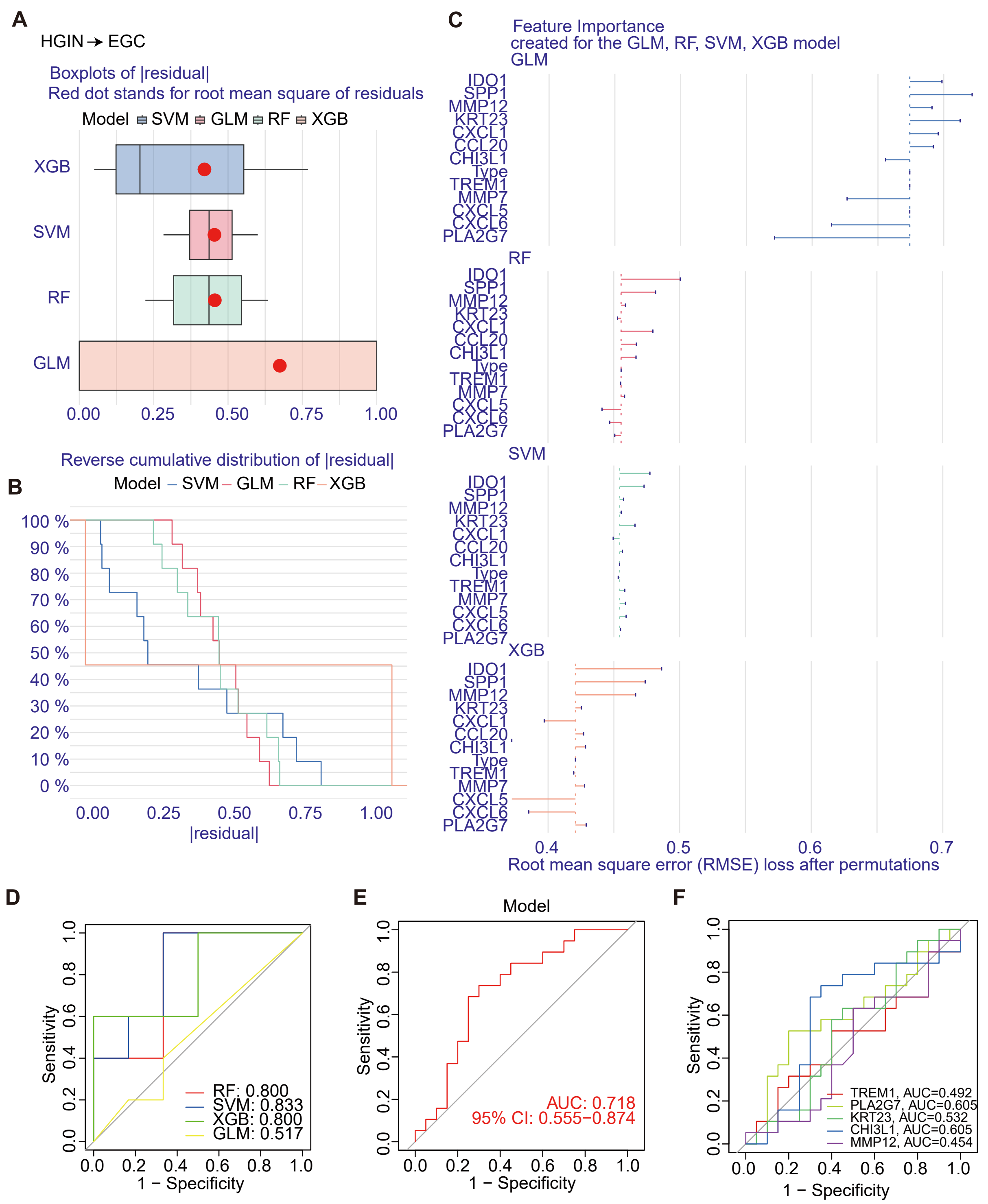

A
HGIN → EGC
Boxplots of |residual|
Red dot stands for root mean square of residuals
Model SVM GLM RF XGB
XGB
SVM
RF
GLM
0.00 0.25 0.50 0.75 1.00
B
Reverse cumulative distribution of |residual|
Model SVM GLM RF XGB
100 % 90 % 80 % 70 % 60 % 50 % 40 % 30 % 20 % 10 % 0 %
0.00 0.25 0.50 0.75 1.00
|residual|
C
Feature Importance
created for the GLM, RF, SVM, XGB model
GLM
RF
SVM
XGB
IDO1
SPP1
MMP12
KRT23
CXCL1
CCL20
CHI3L1
Type
TREM1
MMP7
CXCL5
CXCL6
PLA2G7
0.4 0.5 0.6 0.7
Root mean square error (RMSE) loss after permutations
D
Sensitivity
1 − Specificity
RF: 0.800
SVM: 0.833
XGB: 0.800
GLM: 0.517
E
Model
Sensitivity
1 − Specificity
AUC: 0.718
95% CI: 0.555−0.874
F
Sensitivity
1 − Specificity
TREM1, AUC=0.492
PLA2G7, AUC=0.605
KRT23, AUC=0.532
CHI3L1, AUC=0.605
MMP12, AUC=0.454

Figure S6

A

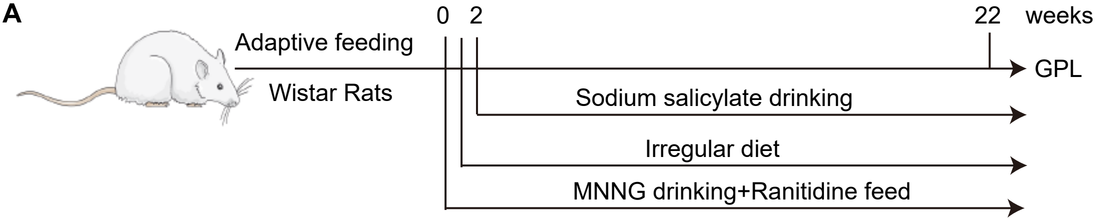
0
2
22
weeks
Adaptive feeding
Wistar Rats
GPL
Sodium salicylate drinking
Irregular diet
MNNG drinking+Ranitidine feed

B

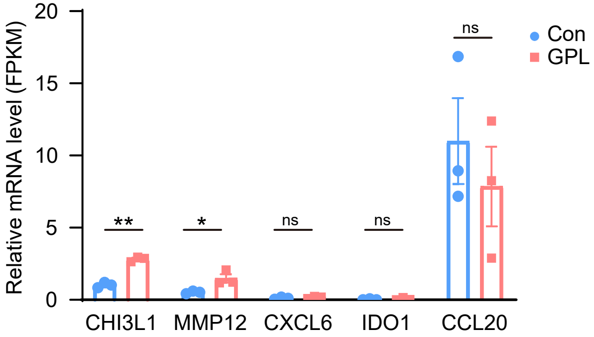
Relative mRNA level (FPKM)
20
15
10
5
0
**
*
ns
ns
ns
Con
GPL
CHI3L1
MMP12
CXCL6
IDO1
CCL20
